# Incidence of mental health diagnoses during the COVID-19 pandemic: a multinational network study

**DOI:** 10.1017/S2045796024000088

**Published:** 2024-03-04

**Authors:** Yi Chai, Kenneth K. C. Man, Hao Luo, Carmen Olga Torre, Yun Kwok Wing, Joseph F. Hayes, David P. J. Osborn, Wing Chung Chang, Xiaoyu Lin, Can Yin, Esther W. Chan, Ivan C. H. Lam, Stephen Fortin, David M. Kern, Dong Yun Lee, Rae Woong Park, Jae-Won Jang, Jing Li, Sarah Seager, Wallis C. Y. Lau, Ian C. K. Wong

**Affiliations:** 1 Centre for Safe Medication Practice and Research, Department of Pharmacology and Pharmacy, LKS Faculty of Medicine, The University of Hong Kong, Hong Kong; 2 The Hong Kong Jockey Club Centre for Suicide Research and Prevention, The University of Hong Kong, Hong Kong; 3 Research Department of Practice and Policy, UCL School of Pharmacy, London, UK; 4 Laboratory of Data Discovery for Health (D^2^4H), Hong Kong Science Park, Hong Kong; 5 Department of Social Work and Social Administration, The University of Hong Kong, Hong Kong; 6 Sau Po Centre on Ageing, The University of Hong Kong, Hong Kong; 7 Real World Data Sciences, Roche, Welwyn Garden City, UK; 8 School of Science and Engineering, University of Groningen, Groningen, The Netherlands; 9 Li Chiu Kong Family Sleep Assessment Unit, Department of Psychiatry, Faculty of Medicine, The Chinese University of Hong Kong, Hong Kong; 10 Division of Psychiatry, University College London, London, UK; 11 Camden and Islington NHS Foundation Trust, London, UK; 12 Department of Psychiatry, School of Clinical Medicine, LKS Faculty of Medicine, The University of Hong Kong, Hong Kong; 13 State Key Laboratory of Brain and Cognitive Sciences, The University of Hong Kong, Hong Kong; 14 Real-World Solutions, IQVIA, Durham, NC, USA; 15 The University of Hong Kong Shenzhen Institute of Research and Innovation, Shenzhen, Guangdong, China; 16 Observation Health Data Analytics, Janssen Research & Development, Titusville, NJ, USA; 17 Department of Epidemiology, Janssen Research & Development, Titusville, NJ, USA; 18 Department of Biomedical Informatics, Ajou University School of Medicine, Suwon, South Korea; 19 Department of Neurology, Kangwon National University Hospital, Kangwon National University School of Medicine, Chuncheon, South Korea

**Keywords:** COVID-19, mental health, OHDSI, OMOP, psychiatric disorder, SARS-CoV-2

## Abstract

**Aims:**

Population-wide restrictions during the COVID-19 pandemic may create barriers to mental health diagnosis. This study aims to examine changes in the number of incident cases and the incidence rates of mental health diagnoses during the COVID-19 pandemic.

**Methods:**

By using electronic health records from France, Germany, Italy, South Korea and the UK and claims data from the US, this study conducted interrupted time-series analyses to compare the monthly incident cases and the incidence of depressive disorders, anxiety disorders, alcohol misuse or dependence, substance misuse or dependence, bipolar disorders, personality disorders and psychoses diagnoses before (January 2017 to February 2020) and after (April 2020 to the latest available date of each database [up to November 2021]) the introduction of COVID-related restrictions.

**Results:**

A total of 629,712,954 individuals were enrolled across nine databases. Following the introduction of restrictions, an immediate decline was observed in the number of incident cases of all mental health diagnoses in the US (rate ratios (RRs) ranged from 0.005 to 0.677) and in the incidence of all conditions in France, Germany, Italy and the US (RRs ranged from 0.002 to 0.422). In the UK, significant reductions were only observed in common mental illnesses. The number of incident cases and the incidence began to return to or exceed pre-pandemic levels in most countries from mid-2020 through 2021.

**Conclusions:**

Healthcare providers should be prepared to deliver service adaptations to mitigate burdens directly or indirectly caused by delays in the diagnosis and treatment of mental health conditions.

## Introduction

As of the end of 2022, there were approximately 0.73 billion confirmed cases of COVID-19, resulting in more than 6 million deaths worldwide (World Health Organization, 2022). The unpredictability and uncertainty of the pandemic itself, along with the policy restrictions and economic recession, may have caused great mental health consequences amongst the global population (Ahmed *et al.*, 2023; Aknin *et al.*, 2022; Moreno *et al.*, 2020). Estimates from a Global Burden of Diseases study showed that the COVID-19 pandemic led to a 27.6% increase in major depressive disorders and a 25.6% increase in anxiety disorders globally (Santomauro *et al.*, 2021). However, a recent meta-analysis of longitudinal studies found no changes in general mental health and anxiety symptoms but a minimal worsening of depression symptoms among the general population during the COVID-19 pandemic (Sun *et al.*, 2023).

Despite the controversy in mental well-being, there are concerns about the paradoxical reduction in mental healthcare provision during the pandemic as healthcare services prioritized COVID-19 cases (Carr *et al.*, 2021). Additionally, containment strategies such as lockdowns and physical distancing, COVID-19-related fears, and worsening financial insecurity may result in the under-diagnosis of mental health conditions (Mansfield *et al.*, 2021; Moreno *et al.*, 2020). Previous studies have reported significant reductions in primary care contacts for patients with mental health conditions during the pandemic compared with pre-pandemic levels in the UK (Carr *et al.*, 2021; Mansfield *et al.*, 2021; Williams *et al.*, 2020). Nevertheless, only a few mental health conditions (i.e., anxiety, depression and eating disorders) or broad categories (i.e., severe mental illness and common mental health problems) were examined in these UK studies over a relatively short follow-up period (up to September 2020). A US study using data from the National Syndromic Surveillance Program also reported a reduction in hospital visits for 10 mental disorders during the pandemic (Anderson *et al.*, 2022). Nonetheless, the study only conducted comparisons between specific periods (i.e., high Delta variant circulation period [July 18 to 14 August 2021] vs. pre-Delta period [April 18 to 15 May 2021]), with the data source limited to emergency departments (Anderson *et al.*, 2022). In contrast, a study in Germany that examined data up to 2020 reported an increase in the diagnosis of anxiety disorders in General Practitioner’s practices between April and December 2020, compared to historical averages (Bohlken *et al.*, 2021). A comprehensive evaluation of the changes in the incidence of multiple mental health diagnoses that includes different healthcare settings and covers sufficiently long observational periods during the pandemic across a number of countries is lacking.

Different countries have heterogeneous healthcare systems, which may have been differentially affected by the pandemic. This may, in turn, impact changes in the incidence of mental health diagnoses. The examination of whether and how the incidence of mental health diagnoses changed during the pandemic across countries could help identify potential gaps in existing mental healthcare systems and guide immediate and longer-term responses. In this population-based multinational network study, we aimed to investigate the changes in the number of incident cases and the incidence rates of seven specific mental health diagnoses during the COVID-19 pandemic in France, Germany, Italy, South Korea, the UK and the US.

## Method

### Data sources

We used data from nine databases, including six electronic health record databases and three claims-based databases. Electronic health record databases consist of IQVIA Longitudinal Patient Database France (France IQVIA) (Jouaville *et al.*, 2015; Kostka *et al.,* 2022), IQVIA Disease Analyser Germany (Germany IQVIA) (Kostka *et al.*, 2022), Longitudinal Patient Database Italy (Italy IQVIA) (Kostka *et al.*, 2022), Ajou University School of Medicine database from South Korea (South Korea AUSOM) (Lai *et al.*, 2018), Kangwon National University database from South Korea (South Korea KUN) and IQVIA Medical Research Data UK (UK IMRD) (Gooden *et al.*, 2022). Claims-based databases were IBM MarketScan Multi-State Medicaid Database US (US MDCD) (Li *et al.*, 2021), IBM MarketScan Medicare Supplemental and Coordination of Benefits Database US (US MDCR) (Li *et al.*, 2021) and IQVIA Open Claims US (US Open Claims) (Kostka *et al.*, 2022). Detailed descriptions of these databases, including their representativeness, are presented in eTable 1 in Supplement and have been previously reported (Luo *et al.* 2023).

All databases were mapped to the Observational Medical Outcomes Partnership (OMOP) Common Data Model (CDM), which ensures that studies could be locally executed at contributing centres in a consistent and standardized manner without modification (Hripcsak *et al.*, 2015). OMOP CDM is maintained by the Observational Health Data Sciences and Informatics (OHDSI) Network, an interdisciplinary initiative applying publicly available data analytic solutions to healthcare databases to improve human health and wellbeing (Hripcsak *et al.*, 2015).

### Study design and participants

The study period was defined as between January 2017 and 1 month before the latest available month for data in 2021 within each database (considering the potential delay in recording relevant diagnoses into systems and the number of records in the latest available month might be underestimated), which varied between databases. The study end date for each database is shown in Table 1. Our current datasets included diagnoses from various visit formats, including inpatient visits, outpatient visits and telehealth consultations. All individuals who received any diagnoses of depressive disorders, anxiety disorders, alcohol misuse or dependence, substance misuse or dependence, bipolar disorders, personality disorders or psychoses (including schizophrenia) during the study period, and had at least 365 days of observation time before the first diagnosis of a mental health condition of interest (index date), were extracted from databases. Only the first diagnosis during the study period was included in the analysis to identify incident cases. A 1-year window prior to the index date was used as a screening period for potentially prevalent cases. All individuals were followed from the index date until the end of continuous enrolment (for UK IMRD, US MDCD and US MDCR) or the last healthcare encounter (for France IQVIA, Germany IQVIA, Italy IQVIA, South Korea AUSOM, South Korea KUN and US Open Claims).

**Table 1.** Total number of unique individuals, incident cases and the incidence of seven mental health diagnoses in each year between 2017 and 2021 in each database

	France IQVIA	Germany IQVIA	Italy IQVIA	South Korea AUSOM	South Korea KUN	UK IMRD	US MDCD	US MDCR	US Open Claims
**Study end date**	July 2021	August 2021	June 2021	June 2021	June 2021	March 2021	May 2021	June 2021	November 2021
**Number of unique individuals**									
2017	5514098	11508048	1372477	585565	178065	4161231	13502346	1400011	387179252
2018	5284256	11266581	1324999	572433	171390	3730732	11510682	1102953	386502702
2019	5036235	10998509	1245660	543472	156206	3551748	12893488	971119	375101559
2020	4408952	9663253	1104118	454761	131147	3108395	12729595	1335986	360037879
2021^a^	3185784	7304254	831007	332459	85845	2573084	12697763	1148670	349621484
**Total**^b^	11569328	23561281	1788262	1091718	280926	5638430	21142977	2882396	561757636
**Number of incident cases (incidence rate, %)**									
**Depressive disorders (%)**									
2017	53507 (0.97)	127902 (1.11)	21846 (1.59)	782 (0.13)	1549 (0.87)	39796 (0.96)	561164 (4.16)	100819 (7.20)	8809768 (2.28)
2018	47901 (0.91)	116408 (1.03)	18073 (1.36)	670 (0.12)	1570 (0.92)	34860 (0.93)	407505 (3.54)	58105 (5.27)	8141375 (2.11)
2019	41607 (0.83)	109627 (1.00)	16323 (1.31)	1044 (0.19)	1385 (0.89)	29817 (0.84)	364772 (2.83)	66130 (6.81)	7101025 (1.89)
2020	39236 (0.89)	105458 (1.09)	12949 (1.17)	774 (0.17)	1381 (1.05)	17144 (0.55)	431182 (3.39)	44458 (3.33)	6210489 (1.72)
2021^a^	26444 (0.83)	70873 (0.97)	7050 (0.85)	282 (0.085)	576 (0.67)	4577 (0.18)	239528 (1.89)	58026 (5.05)	6382863 (1.83)
**Anxiety disorders (%)**									
2017	93626 (1.52)	96637 (0.84)	4557 (0.33)	703 (0.12)	1460 (0.82)	39347 (0.95)	540359 (4.00)	70526 (5.04)	8034240 (2.08)
2018	73140 (1.38)	96113 (0.85)	3807 (0.29)	645 (0.11)	1101 (0.64)	36496 (0.98)	397813 (3.46)	39655 (3.6)	7561607 (1.96)
2019	63794 (1.27)	97412 (0.89)	3454 (0.28)	634 (0.12)	1074 (0.69)	33081 (0.93)	365138 (2.83)	46942 (4.83)	6510047 (1.74)
2020	62627 (1.42)	95067 (0.98)	2946 (0.27)	440 (0.097)	880 (0.67)	18229 (0.59)	450575 (3.54)	32840 (2.46)	6371032 (1.77)
2021^a^	38323 (1.2)	65091 (0.89)	1513 (0.18)	206 (0.062)	397 (0.46)	5485 (0.21)	249104 (1.96)	42794 (3.73)	6649162 (1.90)
**Alcohol misuse or dependence (%)**									
2017	3464 (0.063)	9938 (0.086)	1080 (0.079)	46 (0.0079)	265 (0.15)	4312 (0.10)	90888 (0.67)	6565 (0.47)	1219525 (0.32)
2018	3028 (0.057)	8333 (0.074)	869 (0.066)	28 (0.005)	269 (0.16)	3509 (0.094)	70399 (0.61)	4228 (0.38)	1137421 (0.29)
2019	2722 (0.054)	7993 (0.073)	747 (0.06)	35 (0.006)	242 (0.16)	3052 (0.086)	62619 (0.49)	4723 (0.49)	1034154 (0.28)
2020	2377 (0.054)	7687 (0.08)	570 (0.052)	31 (0.007)	201 (0.15)	2110 (0.068)	88561 (0.7)	3360 (0.25)	932359 (0.26)
2021^a^	1586 (0.05)	4812 (0.066)	317 (0.038)	23 (0.007)	102 (0.12)	609 (0.024)	45784 (0.36)	4594 (0.4)	939671 (0.27)
**Substance misuse or dependence (%)**									
2017	9004 (0.16)	44289 (0.39)	2983 (0.22)	166 (0.028)	322 (0.18)	5922 (0.14)	475904 (3.52)	36761 (2.63)	5464836 (1.41)
2018	8997 (0.17)	39423 (0.35)	2564 (0.19)	112 (0.02)	269 (0.16)	5002 (0.13)	308908 (2.68)	19231 (1.74)	4804573 (1.24)
2019	8146 (0.16)	38974 (0.35)	2134 (0.17)	122 (0.022)	201 (0.13)	4687 (0.13)	252656 (1.96)	21952 (2.26)	4058151 (1.08)
2020	6633 (0.15)	35034 (0.36)	1560 (0.14)	97 (0.021)	182 (0.14)	3205 (0.10)	326431 (2.56)	13972 (1.05)	3299676 (0.92)
2021^a^	4062 (0.13)	23215 (0.32)	755 (0.091)	51 (0.015)	56 (0.065)	946 (0.037)	162917 (1.28)	24206 (2.11)	3198251 (0.92)
**Bipolar disorders (%)**									
2017	2250 (0.041)	2661 (0.023)	654 (0.048)	97 (0.017)	85 (0.048)	896 (0.022)	121357 (0.90)	3629 (0.26)	1035241 (0.27)
2018	1992 (0.038)	2418 (0.022)	617 (0.047)	81 (0.014)	100 (0.058)	712 (0.019)	88788 (0.77)	1988 (0.18)	932519 (0.24)
2019	1658 (0.033)	2003 (0.018)	563 (0.045)	100 (0.018)	85 (0.054)	648 (0.018)	76492 (0.59)	2046 (0.21)	765817 (0.20)
2020	1702 (0.039)	2058 (0.021)	413 (0.037)	76 (0.017)	79 (0.06)	504 (0.016)	91604 (0.72)	1332 (0.10)	697749 (0.19)
2021^a^	988 (0.031)	1397 (0.019)	192 (0.023)	44 (0.013)	16 (0.019)	124 (0.005)	46014 (0.36)	2514 (0.22)	690617 (0.20)
**Personality disorders (%)**									
2017	976 (0.018)	10540 (0.092)	8346 (0.61)	27 (0.0046)	34 (0.019)	1450 (0.035)	44015 (0.33)	1582 (0.11)	263373 (0.068)
2018	898 (0.017)	10603 (0.094)	7553 (0.57)	25 (0.004)	26 (0.015)	1372 (0.037)	36044 (0.31)	934 (0.085)	266674 (0.069)
2019	794 (0.016)	10333 (0.094)	7012 (0.56)	13 (0.002)	28 (0.018)	1311 (0.037)	32909 (0.26)	952 (0.098)	238515 (0.064)
2020	807 (0.018)	9751 (0.10)	4890 (0.44)	15 (0.003)	30 (0.023)	1089 (0.035)	34480 (0.27)	614 (0.046)	216471 (0.06)
2021^a^	525 (0.017)	6500 (0.089)	3063 (0.37)	10 (0.003)	6 (0.007)	271 (0.011)	17952 (0.14)	545 (0.047)	227636 (0.065)
**Psychoses (%)**									
2017	2610 (0.047)	8292 (0.072)	1811 (0.13)	156 (0.027)	320 (0.18)	1923 (0.046)	92678 (0.69)	5798 (0.41)	767311 (0.20)
2018	2319 (0.044)	8088 (0.072)	1482 (0.11)	163 (0.029)	424 (0.25)	1637 (0.044)	70046 (0.61)	2969 (0.27)	693885 (0.18)
2019	1949 (0.039)	7792 (0.071)	1379 (0.11)	141 (0.026)	390 (0.25)	1418 (0.04)	57081 (0.44)	3195 (0.33)	555534 (0.15)
2020	2002 (0.045)	7571 (0.078)	1171 (0.11)	120 (0.026)	365 (0.28)	1198 (0.039)	68654 (0.54)	2299 (0.17)	517644 (0.14)
2021^a^	1139 (0.036)	5010 (0.07)	613 (0.074)	56 (0.017)	174 (0.20)	282 (0.011)	33136 (0.26)	2757 (0.24)	514198 (0.15)

a The full-year data in 2021 were not available at the time of analyses.

b The total number includes all people between January 2016 to the latest available month in 2021 for each database.

### Exposure period and outcome

The exposure started from the introduction of national restrictions and containment strategies in response to the COVID-19 pandemic (Mansfield *et al.*, 2021). According to the Stringency Index developed by the Oxford Coronavirus Government Response Tracker project (Hale *et al.*, 2021), all countries included in our study started introducing strict lockdown measures in March 2020 (eFig. 1 in Supplement) (Luo *et al.* 2023; Hale *et al.*, 2021). We thus divided the study time into three periods: the pre-pandemic period (January 2017 to February 2020), transition period (March 2020) and post-introduction period (April 2020 to the end of the study period).

The outcomes of this study were the changes in the number of incident cases and the incidence rates of the seven mental health diagnoses during the COVID-19 pandemic. Diagnostic codes used for the ascertainment of these mental health conditions were shown in eTable 2 in Supplement.

### Statistical analysis

For each database, the incidence rates for each mental diagnosis between 2017 and 2021 were calculated by dividing the total number of incident cases per month or year by the total number of all unique individuals during the same period. The monthly number of incident cases and the incidence in 2020 and 2021 were compared to 3-year averages in the same months between 2017 and 2019.

Two interrupted time-series analyses were conducted to estimate the impact of the national restrictions and containment strategies on the number of incident cases and the incidence of mental health diagnoses separately, using the quasi-Poisson regression models. Data in the transition period (March 2020) were excluded to account for the time people took to react to the lockdown restrictions (Mansfield *et al.*, 2021). Time (in months) was included as a continuous variable in the model to account for the underlying pre-pandemic trend. Pandemic restriction status was measured by a binary variable (0: pre-pandemic period; 1: post-introduction period) to quantify the immediate change (i.e., level change) in the number of incident cases and the incidence following the introduction of national restrictions and containment strategies. An interaction term between time and pandemic restrictions was used to estimate the gradual change (i.e., slope change) after the restrictions were introduced. All parameters were expressed as rate ratios (RRs) with a 95% confidence interval (CI). The Fourier term was included to control for seasonality and other long-term trends (Bhaskaran *et al.*, 2013; Turner *et al.*, 2021b). The Newey-West standard errors were calculated to correct the possible autocorrelation and possible heteroscedasticity (Turner *et al.*, 2021a, 2021b).

Two sensitivity analyses were conducted by using different transition periods: i) February 2020 and ii) from March to April 2020, to estimate the effect of misclassification of the transition period. All analyses were performed in R software (version 4.1.2) (R Core Team, 2022).

The current study was part of a multinational project entitled ‘Covid-19 pandEmic impacts on mental health Related conditions Via multi-database nEtwork: a LongitudinaL Observational study (CERVELLO)’. The study protocol of CERVELLO was drafted, reviewed and iteratively updated by an international team of academic, clinical and industry stakeholders through the OHDSI network. The protocol and all analytical codes of CERVELLO are publicly available on GitHub (https://github.com/ohdsi-studies/CervelloPrevalence).

### Role of the funding source

The funders of the study had no role in study design, data collection, data analysis, data interpretation or writing of the report.

## Results

A total of 629,712,954 individuals were included in nine databases between 2016 and 2021, ranging from 280,926 individuals in South Korea KUN to 561,757,636 individuals in US Open Claims. Eight of nine databases had a higher proportion of females than males (average proportions of female individuals across the study period ranged from 50.42% in the UK to 57.32% in France), whereas South Korea AUSOM included an average of 49.43% female individuals (eTable 3 in Supplement). Individuals aged below 25 years accounted for 54.96% in US MDCD, and individuals aged 65 years or older accounted for 98.57% in US MDCR, representing the youngest and oldest population among the databases. Regarding the remaining databases, except for the UK and US Open Claims, individuals aged 45–64 years predominated for all databases, ranging from 28.83% in France to 34.88% in South Korea AUSOM.

In most databases, the highest yearly number of incident cases and the incidence were observed in the year 2017 (Table 1). For instance, in France, anxiety disorders had the highest number of incident cases and the incidence (93,626 and 1.52%, respectively) in 2017, while the highest yearly number of incident cases and incidence was observed in depressive disorders in the remaining countries except for South Korea and the UK (Germany: 127,902 [1.11%]; Italy 21,846 [1.59%]; US MDCD: 561,164 [4.16%]; US MDCR: 100,819 [7.20%]; US Open Claims: 8,809,768 [2.28%]).

**Figure 1.**
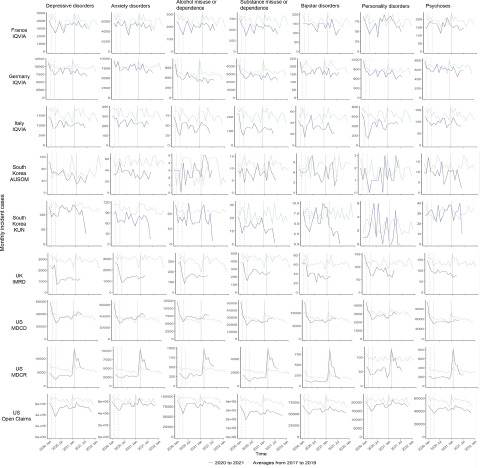
Monthly number of incident cases of seven mental health diagnoses in 2020 and 2021 and historical averages for that month from 2017 to 2019. Vertical dashed lines represent February and April 2020. The vertical solid line represents January 2021.

Figure 1 shows the monthly number of incident cases of seven mental health diagnoses. Compared to the 3-year historical level, we found significant decreases in the number of incident cases of all mental health diagnoses during the initial phase of the pandemic in France, Italy, South Korea, the UK and the US, except for depressive disorders and psychoses in South Korea AUSOM and psychoses in South Korea KUN. After the acute phase of the pandemic, the monthly number of incident cases gradually increased but remained lowered than the 3-year historical level by the end of the study period in most countries. However, in US MDCD and MDCR, the incident cases of most mental health diagnoses recovered to or exceeded pre-pandemic levels.

**Figure 2.**
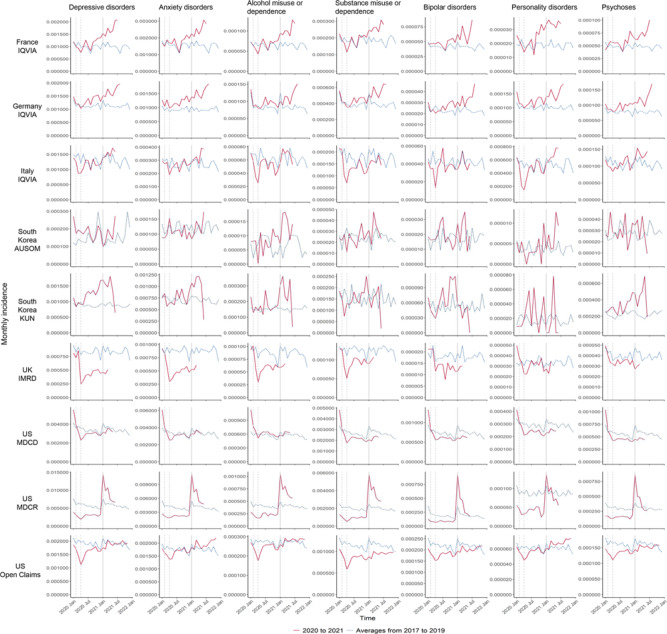
Monthly incidence of seven mental health diagnoses in 2020 and 2021 and historical averages for that month from 2017 to 2019. Vertical dashed lines represent February and April 2020. The vertical solid line represents January 2021.

Figure 2 shows the monthly incidence of seven mental health diagnoses. Similarly, a considerable decline in the incidence of mental health diagnoses was observed during the early stage of the pandemic in France, Italy, South Korea KUN, the UK and the US, with the exception of anxiety and bipolar disorders, and psychoses in France, and depressive disorders, alcohol misuse or dependence, and psychoses in South Korea KUN. There was no reduction in any mental health diagnoses in Germany and South Korea AUSOM. Since mid-2020, the incidence of all diagnoses gradually returned to or surpassed the 3-year historical level, except for some conditions which remained below (i.e., bipolar disorders in South Korea KUN, all conditions except for personality disorders in the UK, all conditions except for depressive and anxiety disorders, and alcohol misuse or dependence in US MDCD, and personality disorders in US MDCR). Patterns varied by country. For example, the incidence of mental health diagnoses in France has returned to the historical level since mid-2020, whilst the increase only became apparent in US MDCR in early 2021.

**Figure 3.**
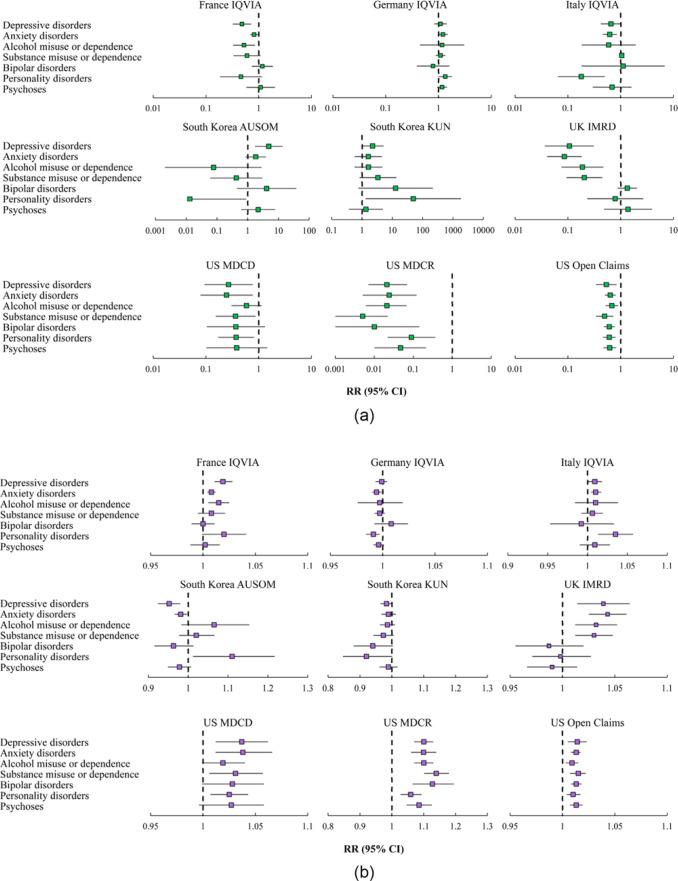
(a) Estimates of the immediate change (i.e., level change) in monthly number of incident cases of seven mental health diagnoses. (b) Estimates of the gradual change (i.e., slope change) in monthly number of incident cases of seven mental health diagnoses.

Figure 3 shows results from interrupted time-series analyses of the monthly number of incident cases of seven mental health diagnoses. Substantial decreases in the number of incident cases were observed immediately following the introduction of restrictions (i.e., level change) in common mental illnesses (i.e., depressive disorders, anxiety disorders, alcohol misuse or dependence and substance misuse or dependence) in the UK and all mental health conditions in the US, ranging from the RR of 0.005 (95% CI, 0.001–0.022) in substance misuse or dependence in US MDCR to 0.677 (0.516–0.889) in alcohol misuse or dependence in US Open Claims (Fig. 3a and eTable 4 in Supplement). All these were followed by a gradual increase (i.e., slope change) after the acute phase of the COVID-19 pandemic (Fig. 3b and eTable 4 in Supplement).

**Figure 4.**
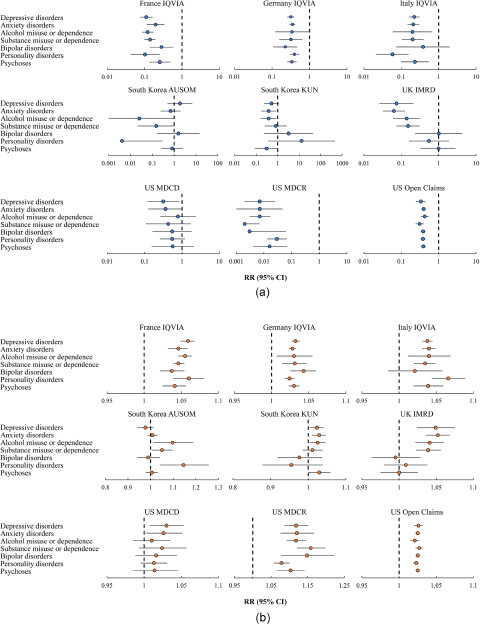
(a) Estimates of the immediate change (i.e., level change) in monthly incidence of seven mental health diagnoses. (b) Estimates of the gradual change (i.e., slope change) in monthly incidence of seven mental health diagnoses.

Results from interrupted time-series analyses of the monthly incidence of seven mental health diagnoses indicated that for most databases, the incidence trends were consistent across mental health conditions (Fig. 4). Substantial immediate decreases in the incidence of all mental health diagnoses were found in all countries except for South Korea. In the UK, a significant decline was also only observed in common mental illnesses. The immediate changes ranged from the RR of 0.002 (95% CI, 0.000–0.007) in substance misuse or dependence in US MDCR to 0.422 (0.328–0.543) in alcohol misuse or dependence in US Open Claims (Fig. 4a and eTable 7 in Supplement). Figure 4b and eTable 7 in Supplement show that the incidence of mental health diagnoses in these databases then increased gradually after the acute phase of the COVID-19 pandemic.

eFigures 2 and 3 show the observed and predicted trends based on the interrupted time-series models of incident cases and the incidence from interrupted series analyses, respectively. Results from sensitivity analyses were generally consistent with our main results (eTables 5–6 and 8–9 in Supplement).

## Discussion

In this multinational network study, we compared the number of incident cases and the incidence of seven mental health diagnoses before and during the COVID-19 pandemic using population-representative electronic health records and claims data from nine databases across six countries. We found that the incident cases and the incidence of mental health diagnoses declined considerably after the introduction of national restrictions and containment strategies, and it gradually returned to or exceeded the pre-pandemic level in 2021 in most countries.

Previous studies reported significant declines in both hospital admissions and emergency department visits in many countries, including the US, the UK, Canada, Italy, Spain, France and Germany, indicating the dynamic adaptation of healthcare systems in response to the COVID-19 pandemic (Di Domenico *et al.*, 2020; Jaehn *et al.*, 2021; Mulholland *et al.*, 2020; Nourazari *et al.*, 2021; Nuñez *et al.*, 2020; Rennert-May *et al.*, 2021; Scaramuzza *et al.*, 2020). From the perspective of mental health conditions, two studies from the UK suggested that the primary care-recorded diagnoses of depression, anxiety disorders and common mental health problems had reduced by 43.0%, 47.8% and 50%, respectively, in April or May 2020 compared to the expected level (Carr *et al.*, 2021; Williams *et al.*, 2020). Another population-based study in the UK found considerable reductions in the incidence of primary care contacts for depression (odds ratio = 0.53), anxiety disorders (0.67) and severe mental illness (0.80) after 29 March 2020 and until 18 July 2020 (Mansfield *et al.*, 2021). Our study not only provides more up-to-date data in the UK but for the first time also examined the number of incident cases and the incidence of diagnoses of mental health conditions during the pandemic in five other countries (i.e., France, Germany, Italy, South Korea and the US). There are some possible explanations for the decline in diagnoses. First, individuals with mild mental health issues might prefer not to visit clinics or hospitals for treatment because of the fear of cross-infection by COVID-19 (Moreno *et al.*, 2020). Second, to reduce COVID-19 transmission, strict infection control strategies were implemented in almost all mental health service premises, including reducing the number of appointments, tightening the admission criteria and treating urgent cases only, which increased barriers to accessing mental health support (Percudani *et al.*, 2020; Sheridan Rains *et al.*, 2021; Xiang *et al.*, 2020). Additionally, it is also possible that individuals have altruistically followed the government’s advice to ‘Stay at Home’ for the broader purpose of the well-being of the community and protecting the healthcare system (Miles *et al.*, 2021). Due to concerns about COVID-19 infection, restrictions on outdoor activities and travelling, and altruism to stay home, individuals with mental health conditions may have opted not to seek mental healthcare during the acute phase of the COVID-19 pandemic.

Although several systematic reviews and meta-analyses have reported that the prevalence of people experiencing symptoms of mental health conditions has increased during the pandemic (Ahmed *et al.*, 2023; Cénat *et al.*, 2021; Troglio da Silva and Neto, 2021; Wu *et al.*, 2021; Xiong *et al.*, 2020), we found substantial reductions in the incident diagnoses of mental health conditions. This might indicate that many people experiencing mental health problems, who would have expected to benefit from receiving mental healthcare, did not access health services during the pandemic. It is reasonable to speculate that there would be an increase in the demand for mental health services following the early stage of this pandemic. This hypothesis was supported by three studies in the UK, which observed increasing trends in mental health condition diagnoses following the acute phase of the pandemic (Carr *et al.*, 2021; Mansfield *et al.*, 2021; Williams *et al.*, 2020). However, the observation period of these studies ended in September 2020, and almost all studies found that the rate of diagnoses of the studied conditions had not returned to the expected (or pre-pandemic) levels. There has been an urgent need for data after September 2020 to comprehensively evaluate the long-term impact of COVID-19 on mental health conditions (Carr *et al.*, 2021). In addition to the potential surge in demand as restrictions were lifted, the unmet need and delays in diagnosis may have exacerbated symptoms of mental health conditions, resulting in an increased risk of self-harm, suicide and other adverse outcomes (Chai *et al.*, 2020; Kisely *et al.*, 2006; Qin, 2011). Our results suggest that there was a sustained period of unmet need during the early phase of the pandemic, and the incident cases and the incidence of mental health diagnoses in most countries continuously increased since mid-2020, often recovering to or exceeding historical averages. As a result of increased incidences of mental health disorders post-pandemic, healthcare systems might be at risk of being overloaded. Findings from the current study should be used by healthcare providers to plan future service provision. Sustained adaptations may be necessary to mitigate the mental healthcare burden that is directly or indirectly caused by delays in diagnoses and treatment of mental health conditions. There should be a focus on the importance of maintaining accessibility to mental health help-seeking and intervention by leveraging technology with tele/digital means and collaborative community support to cope with the abrupt hidden mental problems followed by overcrowding across the acute and post-pandemic period.

To our best knowledge, this study is the first to systematically examine the effect of national COVID-19 restrictions and policies on the diagnoses of a wide range of mental health conditions across countries, with the longest follow-up time since the start of the pandemic. Our study is a collaborative OMOP CDM project in which the same coding and analytical practice enabled rapid and timely investigation of different geographical areas and healthcare systems in a standard and systematic way (Luo *et al.* 2023; Lau *et al.*, 2022). Data from large and diverse databases allowed us to understand how these vulnerable populations were affected by the pandemic across healthcare systems.

This study has limitations. Firstly, the databases included in this research consisted of medical records from different healthcare settings, ranging from primary care to insurance claims. Therefore, the comparison of the absolute incident cases and incidence between different databases or countries should be with caution. However, this would not affect within database comparisons. Secondly, an individual with multiple insurance plans could have multiple identification numbers in the US Open Claims database. This could lead to an overestimation of the total number of patients in the database. However, as our analyses were based on the monthly number of incident cases and incidence, it is unlikely that patients would have multiple insurance plans in the same month. Hence, the effect on the estimation is expected to be minimal. Thirdly, findings generated from some databases may have limited generalizability. For example, South Korean databases only contained data from two hospitals and were more likely to capture people with severe symptoms. The mental health services for these severe cases might have been less affected by the COVID-19 pandemic, which could be a possible explanation for the non-significant reduction in the diagnosis of mental health conditions in South Korea. Further investigations incorporating population-level data sources from South Korea are required to validate our results. Additionally, more than 99% of people in the US MDCR database were aged 45 years and above. However, results from this older population were consistent with that observed in US Open Claims, which consists of all-age patients. Finally, in addition to mental health conditions, the COVID-19 pandemic may have also resulted in a decline in the diagnosis of physical diseases, such as cardiovascular events and metabolic disorders. Further multinational investigation is warranted to explore the changes in the diagnoses of physical diseases during the pandemic.

## Conclusion

There was a reduction in the incident cases and the incidence of mental health diagnoses in many countries immediately after the implementation of stringent national COVID-19 containment strategies. Since the incident cases and the incidence of mental health diagnoses returned to the pre-pandemic level by 2021 in most countries, stakeholders and mental healthcare providers should prepare for potentially delayed but increased demand for care in response to the patients who were not able to access healthcare services for appropriate and timely diagnoses. For example, enhancing population knowledge about mental health and COVID-19 and leveraging technological advances such as telehealth and remote care delivery can help reduce barriers to treatment and improve access to mental health interventions and care. Fostering partnerships between mental health providers, schools, workplaces, community organizations and service users can create a comprehensive mental healthcare network for implementing effective public mental health interventions. Furthermore, the pandemic presents a crucial opportunity to mitigate disparities in previous mental healthcare provision. By prioritizing high-quality and equitable mental healthcare, the current mental healthcare delivery system can be improved to meet the rising demand and future-proof services against further pandemic threats.

## Supporting information

Chai et al. supplementary material Chai et al. supplementary material

## Data Availability

The protocol and analytical codes of this study are publicly available on GitHub (https://github.com/ohdsi-studies/CervelloPrevalence). Data used in this are not publicly available due to restrictions from the data providers.

## Supplementary material

The supplementary material for this article can be found at https://doi.org/10.1017/S2045796024000088.
