# Supplementary material for: Incidence of mental health diagnoses during the COVID-19 pandemic: a multinational network study
Source: Epidemiol Psychiatr Sci. 2024 Mar 4;33:e9. doi: 10.1017/S2045796024000088 (PMC10940053; doi:10.1017/S2045796024000088)
Supplement: Chai et al. supplementary material [file S2045796024000088sup001.docx]

**eTable 1. Descriptions of databases**

| **Database** | **Short name** | **Description** |
| --- | --- | --- |
| IQVIA Longitudinal Patient Database France | France IQVIA | France IQVIA contains anonymized patient records collected from patient management software used by GPs and selected specialists to document patients’ clinical records. Currently, 8.7K providers are contributing to the database covering 17.8M cumulative patients in France. Database limited to 10 years history as per CNIL approval. Observation time is defined by the first and last consultation dates. |
| IQVIA Disease Analyser Germany | Germany IQVIA | Germany IQVIA is comprised of anonymized patient records collected from patient management software used by GPs and selected specialists to document patients’ clinical records. Data coverage includes more than 39.8M cumulative patients in the country and collected from 2.8K providers. Patient visiting more than one provider are not cross-identified for data protection reasons and therefore recorded as separate in the system. Dates of service include from 1992 through present. Observation time is defined by the first and last consultation dates. |
| Longitudinal Patient Database Italy | Italy IQVIA | Italy IQVIA is comprised of anonymized patient records collected from software used by GPs during an office visit to document patients’ clinical records. Data coverage includes about 2.3M cumulative patients with at least one visit provided by 1.4K GPs. Dates of service include from 2011 through present. Observation time is defined by the first and last consultation dates. |
| Ajou University School of Medicine database from South Korea | South Korea AUSOM | South Korea AUSOM contained anonymised patient records that were updated monthly from general hospitals in South Korea. |
| Kangwon National University database from South Korea | South Korea KUN | South Korea KUN contained anonymised patient records that were updated monthly from general hospitals in South Korea. |
| IQVIA Medical Research Data UK | UK IMRD | UK IMRD is a large database of anonymised electronic medical records collected at Primary Care clinics throughout the UK. Data coverage includes 13.7M cumulative patients from 7.4M providers. Dates of service include from 1994 through present. Quality indicators define the start date for that patient (e.g. each patient’s observation period began at the latest of: the patient’s registration date, the acceptable mortality recording date of the practice, the Vision date). The end of the observation period is determined by the end date of registration in the database. |

**eTable 1. *continued.* Descriptions of databases**

| **Database** | **Short name** | **Description** |
| --- | --- | --- |
| Claims-based databases were IBM MarketScan Multi-State Medicaid Database US | US MDCD | US MDCD contains adjudicated US health insurance claims for more than 26 million Medicaid enrollees from multiple states and includes hospital discharge diagnoses, outpatient diagnoses and procedures, and outpatient pharmacy claims as well as ethnicity and Medicare eligibility. Members maintain their same identifier even if they leave the system for a brief period; however the data set lacks lab data. Observation time is defined by the first and last consultation dates. |
| IBM MarketScan Medicare Supplemental and Coordination of Benefits Database US | US MDCR | US MDCR represents health services of approximately 10 million retirees in the United States with primary or Medicare supplemental coverage through privately insured fee-for-service, point-of-service, or capitated health plans. These data include adjudicated health insurance claims (e.g. inpatient, outpatient, and outpatient pharmacy). Additionally, it captures laboratory tests for a subset of the covered lives. Observation time is defined by the first and last consultation dates. |
| IQVIA Open Claims US | US Open Claims | US Open Claims is comprised of pre-adjudicated professional and medical claims at the anonymized patient level collected from office management software and clearinghouse switch sources for the purpose of reimbursement. It includes adjudicated claims for a subset of the medical claims data. The database covers a time period from 2000 to present.11.7M providers are contribution to the database covering 150M active patients (~50% of the US national population). |

**eTable 2.** **Diagnostic codes for mental health conditions identification**

| **Phenotype name** | **Concept ID** | **Concept name** | **Link to ATLAS cohort** |
| --- | --- | --- | --- |
| **Depressive disorders** |  |  | https://atlas-demo.ohdsi.org/#/cohortdefinition/1778278 |
|  | 440383 | Depressive disorders |  |
|  | 35625752 | Depression care management |  |
|  | 44788304 | Depression - enhanced service completed |  |
|  | 4114513 | Depression - motion |  |
|  | 4295031 | Depression management program |  |
|  | 44788282 | Depression - enhanced services administration |  |
|  | 2106310 | Major depressive disorder, severe without psychotic features (MDD) |  |
|  | 2106322 | Major depressive disorder, severe with psychotic features (MDD) |  |
|  | 2106305 | Major depressive disorder, moderate (MDD) |  |
|  | 2106304 | Major depressive disorder, mild (MDD) |  |
|  | 40756912 | Plan for follow-up care for major depressive disorder, documented (MDD ADOL) |  |
| **Anxiety disorders** |  |  | https://atlas-demo.ohdsi.org/#/cohortdefinition/1777957 |
|  | 442077 | Anxiety disorders |  |
|  | 4058397 | Claustrophobia |  |
|  | 4322025 | Mild anxiety |  |
|  | 4214746 | Severe anxiety |  |
|  | 440985 | Phobia |  |
|  | 4087190 | Performance anxiety |  |
|  | 4008683 | Anxiety neurosis |  |
|  | 4263429 | Moderate anxiety |  |
|  | 4338032 | Anxiety hysteria |  |
|  | 4332995 | Needle phobia |  |
|  | 4261239 | Anticipatory anxiety |  |
|  | 4209114 | Phonophobia |  |
|  | 4155074 | School phobia |  |
|  | 4103273 | Recurrent anxiety |  |
|  | 42538968 | Anxiety in pregnancy |  |

**eTable 2. *continued.* Diagnostic codes for mental health conditions identification**

| **Phenotype name** | **Concept ID** | **Concept name** | **Link to ATLAS cohort** |
| --- | --- | --- | --- |
| **Anxiety disorders** |  |  | https://atlas-demo.ohdsi.org/#/cohortdefinition/1777957 |
|  | 4114006 | Parental anxiety |  |
|  | 4102977 | Disturbance of anxiety and fearfulness in childhood and adolescence |  |
|  | 4012101 | Nosophobia |  |
|  | 4112929 | Parasitophobia |  |
| **Alcohol misuse or dependence** |  |  | https://atlas-demo.ohdsi.org/#/cohortdefinition/1777976 |
|  | 433753 | Alcohol abuse |  |
|  | 435243 | Alcohol dependence |  |
|  | 44788279 | Alcohol misuse - enhanced services administration |  |
|  | 44788303 | Alcohol misuse - enhanced service completed |  |
|  | s4218106 | Alcoholism |  |
|  | 439005 | Chronic alcoholism in remission |  |
|  | 436953 | Continuous chronic alcoholism |  |
|  | 4275257 | Detoxication psychiatric therapy for alcoholism |  |
|  | 435532 | Episodic chronic alcoholism |  |
|  | 378726 | Dementia associated with alcoholism |  |
|  | 433735 | Acute alcoholic intoxication in alcoholism |  |
|  | 432609 | Acute alcoholic intoxication in remission, in alcoholism |  |
| **Substance misuse or dependence** |  |  | https://atlas-demo.ohdsi.org/#/cohortdefinition/1777977 |
|  | 4279309 | Substance abuse |  |
|  | 440069 | Drug dependence |  |
|  | 4004672 | Psychoactive substance use disorder |  |
|  | 44786481 | Documentation that patient is a current tobacco user |  |
|  | 4302387 | Substance use treatment: drug withdrawal |  |
|  | 2796056 | Substance Abuse Treatment, Pharmacotherapy |  |
|  | 440787 | Drug dependence in mother complicating pregnancy, childbirth AND/OR puerperium |  |
|  | 434697 | Maternal tobacco abuse |  |
|  | 443274 | Psychostimulant dependence |  |

**eTable 2. *continued.* Diagnostic codes for mental health conditions identification**

| **Phenotype name** | **Concept ID** | **Concept name** | **Link to ATLAS cohort** |
| --- | --- | --- | --- |
| **Substance misuse or dependence** |  |  | https://atlas-demo.ohdsi.org/#/cohortdefinition/1777977 |
|  | 4319165 | Therapeutic drug dependence |  |
|  | 37116660 | Marijuana user |  |
|  | 37116661 | Cocaine user |  |
|  | 4269905 | Referral to drug abuse counsellor |  |
|  | 4217840 | Substance misuse behaviour |  |
|  | 44790195 | Delivery of rehabilitation for drug addiction |  |
|  | 4151569 | Drug addiction notification |  |
|  | 4219382 | Persistent substance misuse |  |
|  | 44787894 | Referral to community drug dependency team |  |
|  | 4149607 | Drug addiction therapy - methadone |  |
| **Bipolar disorders** |  |  | https://atlas-demo.ohdsi.org/#/cohortdefinition/1777958 |
|  | 436665 | Bipolar disorders |  |
|  | 4128935 | Bipolar |  |
| **Personality disorders** |  |  | https://atlas-demo.ohdsi.org/#/cohortdefinition/1777975 |
|  | 441838 | Personality disorders |  |
| **Psychoses** |  |  | https://atlas-demo.ohdsi.org/#/cohortdefinition/1777959 |
|  | 436073 | Psychoses |  |
|  | 4168389 | Borderline schizophrenia |  |
|  | 432590 | Delusional disorder |  |
|  | 439706 | Psychosis with origin in childhood |  |

**eTable 3. Demographic characteristics of individuals in each database between 2017 and 2021**

|  | **Gender** | | | **Age, years** | | | |
| --- | --- | --- | --- | --- | --- | --- | --- |
| **Number of individuals, N (%)** | Female | Male | Unknown | <25 | 25-44 | 45-64 | 65+ |
| **France IQVIA** |  |  |  |  |  |  |  |
| 2017 (n=5514098) | 3152556 (57.17) | 2307275 (41.84) | 54267 (0.98) | 1260674 (22.86) | 1344078 (24.38) | 1591659 (28.87) | 1317687 (23.90) |
| 2018 (n=5284256) | 3024829 (57.24) | 2214876 (41.91) | 44551 (0.84) | 1203238 (22.77) | 1269029 (24.02) | 1521932 (28.80) | 1290057 (24.41) |
| 2019 (n=5036235) | 2887844 (57.34) | 2112766 (41.95) | 35625 (0.71) | 1147263 (22.78) | 1184930 (23.53) | 1440385 (28.60) | 1263657 (25.09) |
| 2020 (n=4408952) | 2527427 (57.32) | 1855352 (42.08) | 26173 (0.59) | 969415 (21.99) | 1030375 (23.37) | 1265652 (28.71) | 1143510 (25.94) |
| 2021 (n= 3185784) | 1837674 (57.68) | 1331902 (41.81) | 16208 (0.51) | 641939 (20.15) | 705441 (22.14) | 935371 (29.36) | 903033 (28.35) |
| Average (n=4685865) | 2686066 (57.32) | 1964434 (41.92) | 35365 (0.76) | 1044506 (22.29) | 1106771 (23.62) | 1351000 (28.83) | 1183588 (25.26) |
| **Germany IQVIA** |  |  |  |  |  |  |  |
| 2017 (n=11508048) | 6535524 (56.79) | 4959853 (43.10) | 12671 (0.11) | 2249075 (19.54) | 2569820 (22.33) | 3623160 (31.48) | 3065993 (26.64) |
| 2018 (n=11266581) | 6375118 (56.58) | 4876729 (43.28) | 14734 (0.13) | 2195232 (19.48) | 2531555 (22.47) | 3512755 (31.18) | 3027039 (26.87) |
| 2019 (n=10998509) | 6188260 (56.26) | 4796603 (43.61) | 13646 (0.12) | 2107681 (19.16) | 2470239 (22.46) | 3405289 (30.96) | 3015300 (27.42) |
| 2020 (n=9663253) | 5433040 (56.22) | 4218680 (43.66) | 11533 (0.12) | 1817336 (18.81) | 2189309 (22.66) | 2981562 (30.85) | 2675046 (27.68) |
| 2021 (n= 7304254) | 4081743 (55.88) | 3214703 (44.01) | 7808 (0.11) | 1322719 (18.11) | 1659328 (22.72) | 2257496 (30.91) | 2064711 (28.27) |
| Average (n=10148129) | 5722737 (56.39) | 4413314 (43.49) | 12078 (0.12) | 1938409 (19.10) | 2284050 (22.51) | 3156052 (31.10) | 2769618 (27.29) |
| **Italy IQVIA** |  |  |  |  |  |  |  |
| 2017 (n=1372477) | 724004 (52.75) | 505495 (36.83) | 142978 (10.42) | 168642 (12.29) | 351951 (25.64) | 457410 (33.33) | 394474 (28.74) |
| 2018 (n=1324999) | 699309 (52.78) | 493807 (37.27) | 131883 (9.95) | 158851 (11.99) | 331695 (25.03) | 447417 (33.77) | 387036 (29.21) |
| 2019 (n=1245660) | 659979 (52.98) | 466166 (37.42) | 119515 (9.59) | 144504 (11.60) | 300956 (24.16) | 423932 (34.03) | 376268 (30.21) |
| 2020 (n=1104118) | 589494 (53.39) | 412807 (37.39) | 101817 (9.22) | 118522 (10.73) | 251529 (22.78) | 380877 (34.50) | 353190 (31.99) |
| 2021 (n= 831007) | 452223 (54.42) | 304884 (36.69) | 73900 (8.89) | 72137 (8.68) | 166806 (20.07) | 291374 (35.06) | 300690 (36.18) |
| Average (n=1175652) | 625002 (53.16) | 436631 (37.14) | 114019 (9.70) | 132531 (11.27) | 280587 (23.87) | 400202 (34.04) | 362332 (30.82) |
| **South Korea AUSOM** |  |  |  |  |  |  |  |
| 2017 (n=585565) | 288146 (49.21) | 297419 (50.79) | - | 133224 (22.75) | 166074 (28.36) | 201047 (34.33) | 85220 (14.55) |
| 2018 (n=572433) | 280996 (49.09) | 291437 (50.91) | - | 126014 (22.01) | 159263 (27.82) | 199352 (34.83) | 87804 (15.34) |
| 2019 (n=543472) | 266521 (49.04) | 276951 (50.96) | - | 115018 (21.16) | 149698 (27.54) | 190939 (35.13) | 87817 (16.16) |
| 2020 (n=454761) | 225910 (49.68) | 228851 (50.32) | - | 86238 (18.96) | 127081 (27.94) | 159185 (35.00) | 82257 (18.09) |
| 2021 (n= 332459) | 168630 (50.72) | 163829 (49.28) | - | 58046 (17.46) | 86395 (25.99) | 117608 (35.38) | 70410 (21.18) |
| Average (n=497738) | 246041 (49.43) | 251697 (50.57) | - | 103708 (20.84) | 137702 (27.67) | 173626 (34.88) | 82702 (16.62) |

**eTable 3. *continued.* Demographic characteristics of individuals in each database between 2017 and 2021**

|  | **Gender** | | | **Age, years** | | | |
| --- | --- | --- | --- | --- | --- | --- | --- |
| **Number of individuals, N (%)** | Female | Male | Unknown | <25 | 25-44 | 45-64 | 65+ |
| **South Korea KUN** |  |  |  |  |  |  |  |
| 2017 (n=178065) | 93548 (52.54) | 84517 (47.46) | - | 40095 (22.52) | 38300 (21.51) | 57365 (32.22) | 42305 (23.76) |
| 2018 (n=171390) | 89969 (52.49) | 81421 (47.51) | - | 37456 (21.85) | 36137 (21.08) | 54946 (32.06) | 42851 (25.00) |
| 2019 (n=156206) | 82385 (52.74) | 73821 (47.26) | - | 32830 (21.02) | 31509 (20.17) | 50099 (32.07) | 41768 (26.74) |
| 2020 (n=131147) | 68823 (52.48) | 62324 (47.52) | - | 24012 (18.31) | 26743 (20.39) | 41574 (31.70) | 38818 (29.60) |
| 2021 (n= 85845) | 45307 (52.78) | 40538 (47.22) | - | 14095 (16.42) | 15538 (18.10) | 26832 (31.26) | 29380 (34.22) |
| Average (n=144531) | 76007 (52.59) | 68524 (47.41) | - | 29698 (20.55) | 29645 (20.51) | 46163 (31.94) | 39025 (27.00) |
| **UK IMRD** |  |  |  |  |  |  |  |
| 2017 (n=4161231) | 2101062 (50.49) | 2060169 (49.51) | - | 1178106 (28.31) | 1142652 (27.46) | 1092156 (26.25) | 748317 (17.98) |
| 2018 (n=3730732) | 1881416 (50.43) | 1849316 (49.57) | - | 1051466 (28.18) | 1028842 (27.58) | 978311 (26.22) | 672113 (18.02) |
| 2019 (n=3551748) | 1791629 (50.44) | 1760119 (49.56) | - | 998264 (28.11) | 975306 (27.46) | 931984 (26.24) | 646194 (18.19) |
| 2020 (n=3108395) | 1566734 (50.40) | 1541661 (49.60) | - | 857882 (27.60) | 852923 (27.44) | 819017 (26.35) | 578573 (18.61) |
| 2021 (n=2573084) | 1293404 (50.27) | 1279680 (49.73) | - | 683529 (26.56) | 699762 (27.20) | 692228 (26.90) | 497565 (19.34) |
| Average (n=3425038) | 1726849 (50.42) | 1698189 (49.58) | - | 953849 (27.85) | 939897 (27.44) | 902739 (26.36) | 628553 (18.35) |
| **US MDCD** |  |  |  |  |  |  |  |
| 2017 (n=13502346) | 7686888 (56.93) | 5815458 (43.07) | - | 7642690 (56.60) | 2863432 (21.21) | 1916829 (14.20) | 1079395 (7.99) |
| 2018 (n=11510682) | 6544142 (56.85) | 4966540 (43.15) | - | 6399022 (55.59) | 2507700 (21.79) | 1689056 (14.67) | 914904 (7.95) |
| 2019 (n=12893488) | 7288900 (56.53) | 5604588 (43.47) | - | 7057403 (54.74) | 2893237 (22.44) | 1925008 (14.93) | 1017840 (7.89) |
| 2020 (n=12729595) | 7182646 (56.42) | 5546949 (43.58) | - | 6898952 (54.20) | 2879637 (22.62) | 1923863 (15.11) | 1027143 (8.07) |
| 2021 (n= 12697763) | 7163095 (56.41) | 5534668 (43.59) | - | 6808892 (53.62) | 2939712 (23.15) | 1913434 (15.07) | 1035725 (8.16) |
| Average (n=12666775) | 7173134 (56.63) | 5493641 (43.37) | - | 6961392 (54.96) | 2816744 (22.24) | 1873638 (14.79) | 1015001 (8.01) |
| **US MDCR** |  |  |  |  |  |  |  |
| 2017 (n=1400011) | 764586 (54.61) | 635425 (45.39) | - | 7 (0.00) | 312 (0.02) | 23426 (1.67) | 1376266 (98.30) |
| 2018 (n=1102953) | 611340 (55.43) | 491613 (44.57) | - | 6 (0.00) | 285 (0.03) | 15452 (1.40) | 1087210 (98.57) |
| 2019 (n=971119) | 539618 (55.57) | 431501 (44.43) | - | 13 (0.00) | 326 (0.03) | 12104 (1.25) | 958676 (98.72) |
| 2020 (n=1335986) | 727681 (54.47) | 608305 (45.53) | - | 19 (0.00) | 467 (0.03) | 20364 (1.52) | 1315136 (98.44) |
| 2021 (n= 1148670) | 623677 (54.30) | 524993 (45.70) | - | 6 (0.00) | 234 (0.02) | 11941 (1.04) | 1136489 (98.94) |
| Average (n=1191748) | 653380 (54.83) | 538368 (45.17) | - | 10 (0.00) | 325 (0.03) | 16657 (1.40) | 1174756 (98.57) |

**eTable 3. *continued.* Demographic characteristics of individuals in each database between 2017 and 2021**

|  | **Gender** | | | **Age, years** | | | |
| --- | --- | --- | --- | --- | --- | --- | --- |
| **Number of individuals, N (%)** | Female | Male | Unknown | <25 | 25-44 | 45-64 | 65+ |
| **US Open Claims** |  |  |  |  |  |  |  |
| 2017 (n=387179252) | 203726907 (52.62) | 182480411 (47.13) | 971934 (0.25) | 124847878 (32.25) | 106826647 (27.59) | 104283316 (26.93) | 51221411 (13.23) |
| 2018 (n=386502702) | 203803137 (52.73) | 181805065 (47.04) | 894500 (0.23) | 121899457 (31.54) | 106810358 (27.64) | 103225800 (26.71) | 54567087 (14.12) |
| 2019 (n=375101559) | 198315819 (52.87) | 175880624 (46.89) | 905116 (0.24) | 114810101 (30.61) | 103136462 (27.50) | 100071153 (26.68) | 57083843 (15.22) |
| 2020 (n=360037879) | 190909484 (53.02) | 168267541 (46.74) | 860854 (0.24) | 105609704 (29.33) | 98862873 (27.46) | 96458353 (26.79) | 59106949 (16.42) |
| 2021 (n= 349621484) | 185716882 (53.12) | 162413975 (46.45) | 1490627 (0.43) | 97893767 (28.00) | 95944359 (27.44) | 93873592 (26.85) | 61909766 (17.71) |
| Average (n=371688575) | 196494446 (52.87) | 174169523 (46.86) | 1024606 (0.27) | 113012181 (30.41) | 102316140 (27.53) | 99582443 (26.79) | 56777811 (15.27) |

**eTable 4.** **Total number of unique individuals, incident cases, and the incidence of seven mental health diagnoses in each year between 2017 and 2021 in each database**

|  | **France IQVIA** | **Germany IQVIA** | **Italy IQVIA** | **South Korea AUSOM** | **South Korea KUN** | | **UK IMRD** | | **US MDCD** | | **US MDCR** | | **US Open Claims** | |  |  |  |  |
| --- | --- | --- | --- | --- | --- | --- | --- | --- | --- | --- | --- | --- | --- | --- | --- | --- | --- | --- |
| **Study end date** | July 2021 | August 2021 | June 2021 | June 2021 | June 2021 | | March 2021 | | May 2021 | | June 2021 | | November 2021 | |  |  |  |  |
| **Number of unique individuals** | | | | | |  | |  | |  | |  | |  | |  |  |  |
| 2017 | 5514098 | 11508048 | 1372477 | 585565 | 178065 | | 4161231 | | 13502346 | | 1400011 | | 387179252 | |  |  |  |  |
| 2018 | 5284256 | 11266581 | 1324999 | 572433 | 171390 | | 3730732 | | 11510682 | | 1102953 | | 386502702 | |  |  |  |  |
| 2019 | 5036235 | 10998509 | 1245660 | 543472 | 156206 | | 3551748 | | 12893488 | | 971119 | | 375101559 | |  |  |  |  |
| 2020 | 4408952 | 9663253 | 1104118 | 454761 | 131147 | | 3108395 | | 12729595 | | 1335986 | | 360037879 | |  |  |  |  |
| 2021^a^ | 3185784 | 7304254 | 831007 | 332459 | 85845 | | 2573084 | | 12697763 | | 1148670 | | 349621484 | |  |  |  |  |
| **Total**^b^ | 11569328 | 23561281 | 1788262 | 1091718 | 280926 | | 5638430 | | 21142977 | | 2882396 | | 561757636 | |  |  |  |  |
| **Number of incident cases (incidence rate, %)** | | | | | |  | |  | |  | |  | |  | |  |  |  |
| **Depressive disorders (%)** | | | | | |  | |  | |  | |  | |  | |  |  |  |
| 2017 | 53507 (0.97) | 127902 (1.11) | 21846 (1.59) | 782 (0.13) | 1549 (0.87) | | 39796 (0.96) | | 561164 (4.16) | | 100819 (7.20) | | 8809768 (2.28) | |  |  |  |  |
| 2018 | 47901 (0.91) | 116408 (1.03) | 18073 (1.36) | 670 (0.12) | 1570 (0.92) | | 34860 (0.93) | | 407505 (3.54) | | 58105 (5.27) | | 8141375 (2.11) | |  |  |  |  |
| 2019 | 41607 (0.83) | 109627 (1.00) | 16323 (1.31) | 1044 (0.19) | 1385 (0.89) | | 29817 (0.84) | | 364772 (2.83) | | 66130 (6.81) | | 7101025 (1.89) | |  |  |  |  |
| 2020 | 39236 (0.89) | 105458 (1.09) | 12949 (1.17) | 774 (0.17) | 1381 (1.05) | | 17144 (0.55) | | 431182 (3.39) | | 44458 (3.33) | | 6210489 (1.72) | |  |  |  |  |
| 2021^a^ | 26444 (0.83) | 70873 (0.97) | 7050 (0.85) | 282 (0.085) | 576 (0.67) | | 4577 (0.18) | | 239528 (1.89) | | 58026 (5.05) | | 6382863 (1.83) | |  |  |  |  |
| **Anxiety disorders (%)** | | | | | |  | |  | |  | |  | |  | |  |  |  |
| 2017 | 93626 (1.52) | 96637 (0.84) | 4557 (0.33) | 703 (0.12) | 1460 (0.82) | | 39347 (0.95) | | 540359 (4.00) | | 70526 (5.04) | | 8034240 (2.08) | |  |  |  |  |
| 2018 | 73140 (1.38) | 96113 (0.85) | 3807 (0.29) | 645 (0.11) | 1101 (0.64) | | 36496 (0.98) | | 397813 (3.46) | | 39655 (3.6) | | 7561607 (1.96) | |  |  |  |  |
| 2019 | 63794 (1.27) | 97412 (0.89) | 3454 (0.28) | 634 (0.12) | 1074 (0.69) | | 33081 (0.93) | | 365138 (2.83) | | 46942 (4.83) | | 6510047 (1.74) | |  |  |  |  |
| 2020 | 62627 (1.42) | 95067 (0.98) | 2946 (0.27) | 440 (0.097) | 880 (0.67) | | 18229 (0.59) | | 450575 (3.54) | | 32840 (2.46) | | 6371032 (1.77) | |  |  |  |  |
| 2021^a^ | 38323 (1.2) | 65091 (0.89) | 1513 (0.18) | 206 (0.062) | 397 (0.46) | | 5485 (0.21) | | 249104 (1.96) | | 42794 (3.73) | | 6649162 (1.90) | |  |  |  |  |
| **Alcohol misuse or dependence (%)** | | | | | |  | |  | |  | |  | |  | |  |  |  |
| 2017 | 3464 (0.063) | 9938 (0.086) | 1080 (0.079) | 46 (0.0079) | 265 (0.15) | | 4312 (0.10) | | 90888 (0.67) | | 6565 (0.47) | | 1219525 (0.32) | |  |  |  |  |
| 2018 | 3028 (0.057) | 8333 (0.074) | 869 (0.066) | 28 (0.005) | 269 (0.16) | | 3509 (0.094) | | 70399 (0.61) | | 4228 (0.38) | | 1137421 (0.29) | |  |  |  |  |
| 2019 | 2722 (0.054) | 7993 (0.073) | 747 (0.06) | 35 (0.006) | 242 (0.16) | | 3052 (0.086) | | 62619 (0.49) | | 4723 (0.49) | | 1034154 (0.28) | |  |  |  |  |
| 2020 | 2377 (0.054) | 7687 (0.08) | 570 (0.052) | 31 (0.007) | 201 (0.15) | | 2110 (0.068) | | 88561 (0.7) | | 3360 (0.25) | | 932359 (0.26) | |  |  |  |  |
| 2021^a^ | 1586 (0.05) | 4812 (0.066) | 317 (0.038) | 23 (0.007) | 102 (0.12) | | 609 (0.024) | | 45784 (0.36) | | 4594 (0.4) | | 939671 (0.27) | |  |  |  |  |
| **Substance misuse or dependence (%)** | | | | | |  | |  | |  | |  | |  | |  |  |  |
| 2017 | 9004 (0.16) | 44289 (0.39) | 2983 (0.22) | 166 (0.028) | 322 (0.18) | | 5922 (0.14) | | 475904 (3.52) | | 36761 (2.63) | | 5464836 (1.41) | |  |  |  |  |
| 2018 | 8997 (0.17) | 39423 (0.35) | 2564 (0.19) | 112 (0.02) | 269 (0.16) | | 5002 (0.13) | | 308908 (2.68) | | 19231 (1.74) | | 4804573 (1.24) | |  |  |  |  |
| 2019 | 8146 (0.16) | 38974 (0.35) | 2134 (0.17) | 122 (0.022) | 201 (0.13) | | 4687 (0.13) | | 252656 (1.96) | | 21952 (2.26) | | 4058151 (1.08) | |  |  |  |  |
| 2020 | 6633 (0.15) | 35034 (0.36) | 1560 (0.14) | 97 (0.021) | 182 (0.14) | | 3205 (0.10) | | 326431 (2.56) | | 13972 (1.05) | | 3299676 (0.92) | |  |  |  |  |
| 2021^a^ | 4062 (0.13) | 23215 (0.32) | 755 (0.091) | 51 (0.015) | 56 (0.065) | | 946 (0.037) | | 162917 (1.28) | | 24206 (2.11) | | 3198251 (0.92) | |  |  |  |  |

**eTable 4. *continued*. Total number of unique individuals, incident cases, and incidence of seven mental health diagnoses in each year between 2017 and 2021 in each database**

|  | **France IQVIA** | **Germany IQVIA** | **Italy IQVIA** | **South Korea ASUOM** | **South Korea KUN** | | **UK IMRD** | | **US MDCD** | | **US MDCR** | | **US Open Claims** | |  |  |  |  |
| --- | --- | --- | --- | --- | --- | --- | --- | --- | --- | --- | --- | --- | --- | --- | --- | --- | --- | --- |
| **Bipolar disorders (%)** | | | | | |  | |  | |  | |  | |  | |  |  |  |
| 2017 | 2250 (0.041) | 2661 (0.023) | 654 (0.048) | 97 (0.017) | 85 (0.048) | | 896 (0.022) | | 121357 (0.90) | | 3629 (0.26) | | 1035241 (0.27) | |  |  |  |  |
| 2018 | 1992 (0.038) | 2418 (0.022) | 617 (0.047) | 81 (0.014) | 100 (0.058) | | 712 (0.019) | | 88788 (0.77) | | 1988 (0.18) | | 932519 (0.24) | |  |  |  |  |
| 2019 | 1658 (0.033) | 2003 (0.018) | 563 (0.045) | 100 (0.018) | 85 (0.054) | | 648 (0.018) | | 76492 (0.59) | | 2046 (0.21) | | 765817 (0.20) | |  |  |  |  |
| 2020 | 1702 (0.039) | 2058 (0.021) | 413 (0.037) | 76 (0.017) | 79 (0.06) | | 504 (0.016) | | 91604 (0.72) | | 1332 (0.10) | | 697749 (0.19) | |  |  |  |  |
| 2021^a^ | 988 (0.031) | 1397 (0.019) | 192 (0.023) | 44 (0.013) | 16 (0.019) | | 124 (0.005) | | 46014 (0.36) | | 2514 (0.22) | | 690617 (0.20) | |  |  |  |  |
| **Personality disorders (%)** | | | | | |  | |  | |  | |  | |  | |  |  |  |
| 2017 | 976 (0.018) | 10540 (0.092) | 8346 (0.61) | 27 (0.0046) | 34 (0.019) | | 1450 (0.035) | | 44015 (0.33) | | 1582 (0.11) | | 263373 (0.068) | |  |  |  |  |
| 2018 | 898 (0.017) | 10603 (0.094) | 7553 (0.57) | 25 (0.004) | 26 (0.015) | | 1372 (0.037) | | 36044 (0.31) | | 934 (0.085) | | 266674 (0.069) | |  |  |  |  |
| 2019 | 794 (0.016) | 10333 (0.094) | 7012 (0.56) | 13 (0.002) | 28 (0.018) | | 1311 (0.037) | | 32909 (0.26) | | 952 (0.098) | | 238515 (0.064) | |  |  |  |  |
| 2020 | 807 (0.018) | 9751 (0.10) | 4890 (0.44) | 15 (0.003) | 30 (0.023) | | 1089 (0.035) | | 34480 (0.27) | | 614 (0.046) | | 216471 (0.06) | |  |  |  |  |
| 2021^a^ | 525 (0.017) | 6500 (0.089) | 3063 (0.37) | 10 (0.003) | 6 (0.007) | | 271 (0.011) | | 17952 (0.14) | | 545 (0.047) | | 227636 (0.065) | |  |  |  |  |
| **Psychoses (%)** | | | | | |  | |  | |  | |  | |  | |  |  |  |
| 2017 | 2610 (0.047) | 8292 (0.072) | 1811 (0.13) | 156 (0.027) | 320 (0.18) | | 1923 (0.046) | | 92678 (0.69) | | 5798 (0.41) | | 767311 (0.20) | |  |  |  |  |
| 2018 | 2319 (0.044) | 8088 (0.072) | 1482 (0.11) | 163 (0.029) | 424 (0.25) | | 1637 (0.044) | | 70046 (0.61) | | 2969 (0.27) | | 693885 (0.18) | |  |  |  |  |
| 2019 | 1949 (0.039) | 7792 (0.071) | 1379 (0.11) | 141 (0.026) | 390 (0.25) | | 1418 (0.04) | | 57081 (0.44) | | 3195 (0.33) | | 555534 (0.15) | |  |  |  |  |
| 2020 | 2002 (0.045) | 7571 (0.078) | 1171 (0.11) | 120 (0.026) | 365 (0.28) | | 1198 (0.039) | | 68654 (0.54) | | 2299 (0.17) | | 517644 (0.14) | |  |  |  |  |
| 2021^a^ | 1139 (0.036) | 5010 (0.07) | 613 (0.074) | 56 (0.017) | 174 (0.20) | | 282 (0.011) | | 33136 (0.26) | | 2757 (0.24) | | 514198 (0.15) | |  |  |  |  |

^a^ The full-year data in 2021 were not available at the time of analyses

^b^ The total number includes all people between January 2016 to the latest available month in 2021 for each database

**eTable 5. Full estimates from interrupted time-series analyses of the monthly number of incident cases of seven mental health diagnoses**

|  | **Background trend (t)** | **Level change** | **Slope change** |
| --- | --- | --- | --- |
|  | **RR (95% CI)** | **RR (95% CI)** | **RR (95% CI)** |
| **France IQVIA** |  |  |  |
| Depressive disorders | 0.989 (0.988-0.991) | 0.480 (0.322-0.714) | 1.019 (1.011-1.028) |
| Anxiety disorders | 0.988 (0.987-0.990) | 0.813 (0.685-0.966) | 1.008 (1.004-1.012) |
| Alcohol misuse or dependence | 0.990 (0.987-0.992) | 0.524 (0.326-0.844) | 1.015 (1.005-1.025) |
| Substance misuse or dependence | 0.995 (0.993-0.996) | 0.597 (0.331-1.077) | 1.008 (0.995-1.021) |
| Bipolar disorders | 0.988 (0.986-0.991) | 1.165 (0.736-1.843) | 1.000 (0.989-1.011) |
| Personality disorders | 0.993 (0.988-0.998) | 0.459 (0.183-1.153) | 1.020 (0.999-1.041) |
| Psychoses | 0.988 (0.984-0.991) | 1.082 (0.581-2.015) | 1.002 (0.988-1.016) |
| **Germany IQVIA** |  |  |  |
| Depressive disorders | 0.993 (0.993-0.994) | 1.086 (0.828-1.426) | 0.999 (0.993-1.004) |
| Anxiety disorders | 1.000 (0.999-1.001) | 1.209 (0.977-1.495) | 0.994 (0.990-0.999) |
| Alcohol misuse or dependence | 0.991 (0.985-0.996) | 1.162 (0.443-3.051) | 0.997 (0.976-1.019) |
| Substance misuse or dependence | 0.994 (0.990-0.998) | 1.087 (0.876-1.347) | 0.997 (0.992-1.002) |
| Bipolar disorders | 0.989 (0.985-0.993) | 0.784 (0.387-1.590) | 1.008 (0.992-1.024) |
| Personality disorders | 0.999 (0.998-1.001) | 1.339 (0.998-1.796) | 0.991 (0.984-0.997) |
| Psychoses | 0.997 (0.996-0.998) | 1.166 (0.939-1.448) | 0.996 (0.991-1.001) |
| **Italy IQVIA** |  |  |  |
| Depressive disorders | 0.987 (0.985-0.990) | 0.649 (0.419-1.005) | 1.009 (1.000-1.018) |
| Anxiety disorders | 0.988 (0.985-0.991) | 0.615 (0.451-0.838) | 1.010 (1.004-1.017) |
| Alcohol misuse or dependence | 0.985 (0.980-0.990) | 0.589 (0.181-1.917) | 1.010 (0.984-1.038) |
| Substance misuse or dependence | 0.587 (0.304-1.132) | 1.040 (0.993-1.090) | 1.006 (0.992-1.019) |
| Bipolar disorders | 0.992 (0.987-0.997) | 1.104 (0.180-6.777) | 0.992 (0.953-1.033) |
| Personality disorders | 0.992 (0.989-0.994) | 0.178 (0.064-0.495) | 1.035 (1.013-1.057) |
| Psychoses | 0.988 (0.984-0.992) | 0.682 (0.293-1.590) | 1.009 (0.990-1.028) |
| **South Korea AUSOM** |  |  |  |
| Depressive disorders | 1.014 (1.001-1.028) | 4.801 (1.704-13.523) | 0.952 (0.924-0.980) |
| Anxiety disorders | 0.993 (0.989-0.998) | 1.807 (0.843-3.875) | 0.981 (0.966-0.997) |
| Alcohol misuse or dependence | 0.988 (0.969-1.006) | 0.077 (0.002-2.786) | 1.065 (0.983-1.153) |
| Substance misuse or dependence | 0.986 (0.977-0.995) | 0.422 (0.060-2.954) | 1.020 (0.977-1.066) |
| Bipolar disorders | 1.002 (0.991-1.013) | 4.093 (0.443-37.800) | 0.963 (0.915-1.013) |
| Personality disorders | 0.976 (0.958-0.995) | 0.013 (0.000-0.879) | 1.110 (1.012-1.217) |
| Psychoses | 0.995 (0.992-0.998) | 2.172 (0.607-7.769) | 0.978 (0.949-1.007) |
| **South Korea KUN** |  |  |  |
| Depressive disorders | 0.994 (0.990-0.998) | 2.207 (0.952-5.118) | 0.983 (0.964-1.002) |
| Anxiety disorders | 0.987 (0.983-0.991) | 1.591 (0.567-4.465) | 0.989 (0.967-1.012) |
| Alcohol misuse or dependence | 0.996 (0.992-1.000) | 1.607 (0.556-4.651) | 0.986 (0.962-1.010) |
| Substance misuse or dependence | 0.982 (0.979-0.985) | 3.302 (0.815-13.374) | 0.973 (0.942-1.005) |
| Bipolar disorders | 0.996 (0.986-1.006) | 12.695 (0.750-214.832) | 0.940 (0.880-1.003) |
| Personality disorders | 0.987 (0.975-1.000) | 48.539 (1.302-1810.263) | 0.920 (0.846-1.001) |
| Psychoses | 1.006 (0.999-1.013) | 1.336 (0.368-4.845) | 0.989 (0.961-1.018) |
| **UK IMRD** |  |  |  |
| Depressive disorders | 0.987 (0.985-0.990) | 0.106 (0.036-0.307) | 1.039 (1.014-1.064) |
| Anxiety disorders | 0.992 (0.990-0.994) | 0.085 (0.040-0.181) | 1.043 (1.025-1.061) |
| Alcohol misuse or dependence | 0.986 (0.983-0.990) | 0.187 (0.075-0.466) | 1.032 (1.012-1.052) |
| Substance misuse or dependence | 0.990 (0.988-0.992) | 0.203 (0.093-0.443) | 1.030 (1.012-1.048) |
| Bipolar disorders | 0.988 (0.984-0.992) | 1.325 (0.869-2.021) | 0.987 (0.955-1.020) |
| Personality disorders | 0.997 (0.993-1.001) | 0.781 (0.231-2.639) | 0.998 (0.971-1.027) |
| Psychoses | 0.988 (0.985-0.991) | 1.371 (0.481-3.908) | 0.990 (0.966-1.014) |
| **US MDCD** |  |  |  |
| Depressive disorders | 0.987 (0.982-0.992) | 0.267 (0.093-0.769) | 1.037 (1.012-1.062) |
| Anxiety disorders | 0.989 (0.984-0.995) | 0.244 (0.078-0.769) | 1.038 (1.012-1.066) |
| Alcohol misuse or dependence | 0.992 (0.982-1.002) | 0.584 (0.302-1.132) | 1.019 (0.999-1.040) |
| Substance misuse or dependence | 0.981 (0.970-0.992) | 0.362 (0.152-0.867) | 1.031 (1.006-1.057) |
| Bipolar disorders | 0.986 (0.980-0.992) | 0.367 (0.103-1.310) | 1.028 (0.999-1.058) |
| Personality disorders | 0.991 (0.987-0.994) | 0.371 (0.169-0.815) | 1.025 (1.007-1.043) |
| Psychoses | 0.986 (0.980-0.992) | 0.381 (0.101-1.441) | 1.027 (0.996-1.058) |
| **US MDCR** |  |  |  |
| Depressive disorders | 0.980 (0.973-0.988) | 0.021 (0.007-0.069) | 1.100 (1.070-1.129) |
| Anxiety disorders | 0.980 (0.972-0.988) | 0.024 (0.005-0.121) | 1.099 (1.060-1.139) |
| Alcohol misuse or dependence | 0.985 (0.980-0.990) | 0.021 (0.006-0.067) | 1.100 (1.070-1.130) |
| Substance misuse or dependence | 0.977 (0.971-0.983) | 0.005 (0.001-0.022) | 1.139 (1.101-1.179) |
| Bipolar disorders | 0.973 (0.961-0.986) | 0.010 (0.001-0.141) | 1.127 (1.065-1.194) |
| Personality disorders | 0.979 (0.973-0.985) | 0.089 (0.022-0.365) | 1.059 (1.027-1.093) |
| Psychoses | 0.974 (0.968-0.980) | 0.047 (0.010-0.212) | 1.085 (1.046-1.125) |
| **US Open Claims** |  |  |  |
| Depressive disorders | 0.991 (0.990-0.992) | 0.531 (0.339-0.831) | 1.014 (1.005-1.023) |
| Anxiety disorders | 0.992 (0.990-0.993) | 0.630 (0.498-0.797) | 1.013 (1.008-1.017) |
| Alcohol misuse or dependence | 0.993 (0.991-0.995) | 0.677 (0.516-0.889) | 1.009 (1.003-1.015) |
| Substance misuse or dependence | 0.988 (0.987-0.989) | 0.493 (0.337-0.721) | 1.015 (1.007-1.022) |
| Bipolar disorders | 0.988 (0.986-0.989) | 0.605 (0.477-0.768) | 1.013 (1.008-1.018) |
| Personality disorders | 0.996 (0.994-0.998) | 0.604 (0.458-0.797) | 1.010 (1.004-1.017) |
| Psychoses | 0.987 (0.986-0.989) | 0.616 (0.463-0.820) | 1.013 (1.007-1.019) |

**eTable 6. Full estimates from sensitivity analysis one: interrupted time-series analyses of the monthly number of incident cases of seven mental health diagnoses using February 2020 as the transition period**

|  | **Background trend (t)** | **Level change** | **Slope change** |
| --- | --- | --- | --- |
|  | **RR (95% CI)** | **RR (95% CI)** | **RR (95% CI)** |
| **France IQVIA** |  |  |  |
| Depressive disorders | 0.989 (0.988-0.991) | 0.448 (0.314-0.638) | 1.021 (1.013-1.028) |
| Anxiety disorders | 0.989 (0.987-0.990) | 0.891 (0.771-1.028) | 1.006 (1.002-1.010) |
| Alcohol misuse or dependence | 0.990 (0.987-0.992) | 0.475 (0.312-0.722) | 1.017 (1.009-1.026) |
| Substance misuse or dependence | 0.995 (0.994-0.997) | 0.613 (0.399-0.943) | 1.007 (0.997-1.017) |
| Bipolar disorders | 0.987 (0.985-0.990) | 1.111 (0.867-1.423) | 1.001 (0.995-1.007) |
| Personality disorders | 0.992 (0.987-0.998) | 0.422 (0.184-0.970) | 1.022 (1.003-1.042) |
| Psychoses | 0.987 (0.984-0.991) | 1.167 (0.669-2.035) | 1.001 (0.988-1.014) |
| **Germany IQVIA** |  |  |  |
| Depressive disorders | 0.994 (0.993-0.995) | 1.054 (0.837-1.326) | 0.999 (0.994-1.004) |
| Anxiety disorders | 0.994 (0.993-0.994) | 1.054 (0.937-1.185) | 0.999 (0.997-1.002) |
| Alcohol misuse or dependence | 0.991 (0.985-0.996) | 1.190 (0.497-2.852) | 0.996 (0.977-1.016) |
| Substance misuse or dependence | 0.994 (0.990-0.998) | 1.068 (0.888-1.284) | 0.997 (0.993-1.001) |
| Bipolar disorders | 0.989 (0.985-0.993) | 0.806 (0.424-1.531) | 1.007 (0.993-1.022) |
| Personality disorders | 1.000 (0.999-1.001) | 1.328 (1.101-1.602) | 0.990 (0.986-0.995) |
| Psychoses | 0.997 (0.995-0.999) | 1.144 (0.877-1.493) | 0.996 (0.991-1.002) |
| **Italy IQVIA** |  |  |  |
| Depressive disorders | 0.987 (0.985-0.990) | 0.481 (0.280-0.827) | 1.015 (1.004-1.027) |
| Anxiety disorders | 0.988 (0.985-0.991) | 0.726 (0.597-0.883) | 1.007 (1.002-1.012) |
| Alcohol misuse or dependence | 0.984 (0.979-0.990) | 0.431 (0.145-1.284) | 1.017 (0.992-1.043) |
| Substance misuse or dependence | 0.986 (0.984-0.989) | 0.438 (0.225-0.855) | 1.013 (0.998-1.027) |
| Bipolar disorders | 0.993 (0.988-0.997) | 1.081 (0.274-4.262) | 0.992 (0.963-1.022) |
| Personality disorders | 0.992 (0.989-0.995) | 0.115 (0.037-0.358) | 1.044 (1.020-1.069) |
| Psychoses | 0.988 (0.984-0.992) | 0.686 (0.321-1.467) | 1.009 (0.991-1.027) |
| **South Korea AUSOM** |  |  |  |
| Depressive disorders | 1.015 (1.000-1.031) | 6.499 (1.962-21.526) | 0.945 (0.913-0.978) |
| Anxiety disorders | 0.994 (0.991-0.998) | 1.626 (0.939-2.816) | 0.982 (0.971-0.994) |
| Alcohol misuse or dependence | 0.987 (0.968-1.006) | 0.124 (0.005-3.067) | 1.055 (0.981-1.135) |
| Substance misuse or dependence | 0.987 (0.977-0.996) | 0.592 (0.104-3.357) | 1.013 (0.973-1.054) |
| Bipolar disorders | 1.003 (0.992-1.015) | 3.020 (0.411-22.178) | 0.968 (0.925-1.014) |
| Personality disorders | 0.976 (0.957-0.996) | 0.049 (0.001-1.976) | 1.079 (0.993-1.172) |
| Psychoses | 0.996 (0.992-1.000) | 5.170 (1.555-17.185) | 0.959 (0.932-0.986) |
| **South Korea KUN** |  |  |  |
| Depressive disorders | 0.995 (0.990-0.999) | 1.796 (0.844-3.821) | 0.987 (0.970-1.004) |
| Anxiety disorders | 0.987 (0.983-0.991) | 1.046 (0.398-2.750) | 0.998 (0.977-1.020) |
| Alcohol misuse or dependence | 0.998 (0.993-1.003) | 1.544 (0.696-3.422) | 0.985 (0.967-1.004) |
| Substance misuse or dependence | 0.981 (0.978-0.985) | 1.512 (0.300-7.609) | 0.990 (0.953-1.028) |
| Bipolar disorders | 0.997 (0.985-1.008) | 6.727 (0.485-93.289) | 0.952 (0.896-1.011) |
| Personality disorders | 0.987 (0.966-1.009) | 17.698 (0.471-664.973) | 0.941 (0.863-1.026) |
| Psychoses | 1.007 (1.000-1.014) | 1.393 (0.442-4.384) | 0.988 (0.962-1.014) |
| **UK IMRD** |  |  |  |
| Depressive disorders | 0.988 (0.984-0.992) | 0.746 (0.211-2.642) | 0.995 (0.965-1.025) |
| Anxiety disorders | 0.992 (0.989-0.995) | 0.243 (0.091-0.649) | 1.019 (0.996-1.042) |
| Alcohol misuse or dependence | 0.985 (0.981-0.989) | 0.240 (0.076-0.753) | 1.027 (1.000-1.055) |
| Substance misuse or dependence | 0.990 (0.987-0.992) | 0.234 (0.146-0.377) | 1.027 (1.016-1.039) |
| Bipolar disorders | 0.987 (0.983-0.992) | 2.340 (0.704-7.778) | 0.975 (0.948-1.004) |
| Personality disorders | 0.997 (0.993-1.002) | 1.046 (0.360-3.037) | 0.992 (0.968-1.017) |
| Psychoses | 0.988 (0.984-0.991) | 1.266 (0.508-3.154) | 0.992 (0.970-1.013) |
| **US MDCD** |  |  |  |
| Depressive disorders | 0.985 (0.980-0.990) | 0.256 (0.104-0.632) | 1.039 (1.018-1.061) |
| Anxiety disorders | 0.988 (0.982-0.993) | 0.295 (0.109-0.801) | 1.036 (1.012-1.060) |
| Alcohol misuse or dependence | 0.989 (0.983-0.995) | 0.580 (0.218-1.544) | 1.021 (0.998-1.045) |
| Substance misuse or dependence | 0.978 (0.972-0.985) | 0.363 (0.103-1.284) | 1.033 (1.004-1.064) |
| Bipolar disorders | 0.984 (0.979-0.990) | 0.453 (0.150-1.371) | 1.025 (0.999-1.051) |
| Personality disorders | 0.990 (0.986-0.993) | 0.468 (0.235-0.931) | 1.021 (1.005-1.037) |
| Psychoses | 0.984 (0.978-0.990) | 0.467 (0.146-1.497) | 1.023 (0.996-1.051) |
| **US MDCR** |  |  |  |
| Depressive disorders | 0.981 (0.976-0.987) | 0.021 (0.007-0.062) | 1.099 (1.071-1.128) |
| Anxiety disorders | 0.982 (0.976-0.988) | 0.029 (0.010-0.079) | 1.093 (1.066-1.121) |
| Alcohol misuse or dependence | 0.986 (0.981-0.991) | 0.020 (0.007-0.054) | 1.099 (1.074-1.126) |
| Substance misuse or dependence | 0.978 (0.972-0.985) | 0.005 (0.002-0.018) | 1.137 (1.104-1.171) |
| Bipolar disorders | 0.975 (0.962-0.987) | 0.010 (0.001-0.097) | 1.127 (1.071-1.187) |
| Personality disorders | 0.979 (0.973-0.986) | 0.076 (0.021-0.276) | 1.063 (1.032-1.094) |
| Psychoses | 0.974 (0.968-0.981) | 0.042 (0.011-0.162) | 1.087 (1.051-1.123) |
| **US Open Claims** |  |  |  |
| Depressive disorders | 0.991 (0.990-0.993) | 0.511 (0.345-0.757) | 1.015 (1.006-1.023) |
| Anxiety disorders | 0.992 (0.990-0.993) | 0.606 (0.487-0.753) | 1.013 (1.009-1.018) |
| Alcohol misuse or dependence | 0.993 (0.992-0.995) | 0.636 (0.462-0.875) | 1.010 (1.003-1.016) |
| Substance misuse or dependence | 0.988 (0.987-0.989) | 0.502 (0.374-0.673) | 1.014 (1.008-1.020) |
| Bipolar disorders | 0.988 (0.986-0.990) | 0.589 (0.477-0.728) | 1.013 (1.009-1.018) |
| Personality disorders | 0.996 (0.994-0.998) | 0.593 (0.465-0.755) | 1.011 (1.005-1.017) |
| Psychoses | 0.987 (0.985-0.989) | 0.595 (0.459-0.771) | 1.014 (1.008-1.019) |

**eTable 7. Full estimates from sensitivity analysis two: interrupted time-series analyses of the monthly number of incident cases of seven mental health diagnoses using March to April 2022 as the transition period**

|  | **Background trend (t)** | **Level change** | **Slope change** |
| --- | --- | --- | --- |
|  | **RR (95% CI)** | **RR (95% CI)** | **RR (95% CI)** |
| **France IQVIA** |  |  |  |
| Depressive disorders | 0.989 (0.988-0.991) | 0.723 (0.611-0.854) | 1.010 (1.007-1.014) |
| Anxiety disorders | 0.988 (0.987-0.990) | 0.901 (0.726-1.117) | 1.006 (1.001-1.011) |
| Alcohol misuse or dependence | 0.990 (0.987-0.992) | 0.855 (0.674-1.084) | 1.005 (0.999-1.011) |
| Substance misuse or dependence | 0.995 (0.994-0.996) | 0.978 (0.753-1.271) | 0.997 (0.991-1.003) |
| Bipolar disorders | 0.988 (0.986-0.990) | 1.270 (1.002-1.609) | 0.998 (0.992-1.004) |
| Personality disorders | 0.993 (0.990-0.995) | 0.749 (0.362-1.550) | 1.010 (0.994-1.026) |
| Psychoses | 0.988 (0.984-0.991) | 1.044 (0.526-2.073) | 1.003 (0.987-1.018) |
| **Germany IQVIA** |  |  |  |
| Depressive disorders | 0.994 (0.991-0.996) | 1.334 (0.805-2.209) | 0.994 (0.983-1.005) |
| Anxiety disorders | 1.000 (0.997-1.002) | 1.384 (0.858-2.233) | 0.991 (0.981-1.002) |
| Alcohol misuse or dependence | 0.991 (0.986-0.996) | 1.484 (0.542-4.059) | 0.992 (0.969-1.014) |
| Substance misuse or dependence | 0.994 (0.990-0.998) | 1.263 (0.607-2.628) | 0.994 (0.977-1.010) |
| Bipolar disorders | 0.989 (0.985-0.993) | 0.932 (0.436-1.991) | 1.004 (0.987-1.022) |
| Personality disorders | 0.999 (0.996-1.002) | 1.566 (0.878-2.792) | 0.987 (0.974-1.000) |
| Psychoses | 0.997 (0.994-1.000) | 1.294 (0.717-2.335) | 0.994 (0.981-1.007) |
| **Italy IQVIA** |  |  |  |
| Depressive disorders | 0.987 (0.985-0.990) | 1.027 (0.838-1.259) | 0.998 (0.994-1.003) |
| Anxiety disorders | 0.988 (0.985-0.991) | 0.923 (0.700-1.217) | 1.001 (0.995-1.008) |
| Alcohol misuse or dependence | 0.985 (0.980-0.989) | 1.200 (0.362-3.976) | 0.995 (0.968-1.022) |
| Substance misuse or dependence | 0.988 (0.983-0.992) | 1.303 (0.397-4.279) | 0.988 (0.962-1.015) |
| Bipolar disorders | 0.992 (0.987-0.998) | 3.478 (0.902-13.419) | 0.967 (0.938-0.998) |
| Personality disorders | 0.992 (0.990-0.994) | 0.453 (0.278-0.740) | 1.014 (1.003-1.026) |
| Psychoses | 0.988 (0.984-0.992) | 0.854 (0.340-2.142) | 1.004 (0.983-1.025) |
| **South Korea AUSOM** |  |  |  |
| Depressive disorders | 1.014 (0.999-1.030) | 3.804 (1.318-10.983) | 0.956 (0.929-0.983) |
| Anxiety disorders | 0.993 (0.988-0.998) | 0.872 (0.566-1.344) | 0.996 (0.985-1.007) |
| Alcohol misuse or dependence | 0.988 (0.970-1.006) | 0.248 (0.006-9.689) | 1.040 (0.957-1.130) |
| Substance misuse or dependence | 0.987 (0.979-0.995) | 0.842 (0.200-3.546) | 1.005 (0.970-1.041) |
| Bipolar disorders | 1.002 (0.991-1.013) | 5.975 (0.517-69.002) | 0.954 (0.902-1.010) |
| Personality disorders | 0.976 (0.957-0.995) | 0.009 (0.000-0.967) | 1.119 (1.010-1.239) |
| Psychoses | 0.995 (0.992-0.997) | 1.679 (0.402-7.013) | 0.983 (0.951-1.016) |
| **South Korea KUN** |  |  |  |
| Depressive disorders | 0.994 (0.990-0.998) | 3.214 (1.310-7.881) | 0.975 (0.955-0.995) |
| Anxiety disorders | 0.987 (0.982-0.991) | 1.575 (0.497-4.988) | 0.989 (0.964-1.016) |
| Alcohol misuse or dependence | 0.996 (0.992-1.000) | 2.068 (0.605-7.074) | 0.980 (0.952-1.009) |
| Substance misuse or dependence | 0.982 (0.976-0.987) | 4.647 (0.623-34.675) | 0.965 (0.920-1.011) |
| Bipolar disorders | 0.996 (0.986-1.006) | 40.186 (2.181-740.583) | 0.915 (0.860-0.973) |
| Personality disorders | 0.988 (0.982-0.994) | 309.860 (51.543-1862.772) | 0.881 (0.845-0.920) |
| Psychoses | 1.006 (0.999-1.013) | 1.135 (0.271-4.747) | 0.992 (0.961-1.025) |
| **UK IMRD** |  |  |  |
| Depressive disorders | 0.987 (0.985-0.989) | 0.223 (0.130-0.383) | 1.022 (1.009-1.035) |
| Anxiety disorders | 0.992 (0.990-0.994) | 0.145 (0.084-0.249) | 1.031 (1.018-1.044) |
| Alcohol misuse or dependence | 0.986 (0.983-0.990) | 0.452 (0.281-0.728) | 1.012 (1.001-1.023) |
| Substance misuse or dependence | 0.990 (0.988-0.992) | 0.427 (0.299-0.611) | 1.013 (1.005-1.022) |
| Bipolar disorders | 0.988 (0.984-0.992) | 1.290 (0.281-5.922) | 0.987 (0.953-1.023) |
| Personality disorders | 0.997 (0.993-1.001) | 1.250 (0.327-4.780) | 0.988 (0.957-1.019) |
| Psychoses | 0.988 (0.985-0.991) | 1.595 (0.484-5.251) | 0.986 (0.959-1.014) |
| **US MDCD** |  |  |  |
| Depressive disorders | 0.987 (0.982-0.992) | 0.377 (0.115-1.243) | 1.029 (1.002-1.058) |
| Anxiety disorders | 0.990 (0.984-0.995) | 0.399 (0.107-1.487) | 1.028 (0.997-1.059) |
| Alcohol misuse or dependence | 0.992 (0.982-1.002) | 0.792 (0.391-1.603) | 1.013 (0.991-1.035) |
| Substance misuse or dependence | 0.515 (0.207-1.286) | 0.515 (0.207-1.286) | 1.023 (0.997-1.051) |
| Bipolar disorders | 0.986 (0.980-0.992) | 0.399 (0.097-1.636) | 1.026 (0.994-1.060) |
| Personality disorders | 0.991 (0.986-0.996) | 0.503 (0.325-0.779) | 1.018 (1.006-1.031) |
| Psychoses | 0.986 (0.980-0.992) | 0.421 (0.096-1.841) | 1.025 (0.991-1.060) |
| **US MDCR** |  |  |  |
| Depressive disorders | 0.981 (0.975-0.986) | 0.031 (0.008-0.113) | 1.093 (1.060-1.127) |
| Anxiety disorders | 0.981 (0.975-0.987) | 0.036 (0.009-0.138) | 1.090 (1.056-1.125) |
| Alcohol misuse or dependence | 0.985 (0.980-0.990) | 0.026 (0.007-0.094) | 1.096 (1.064-1.129) |
| Substance misuse or dependence | 0.977 (0.971-0.983) | 0.007 (0.001-0.035) | 1.133 (1.092-1.175) |
| Bipolar disorders | 0.973 (0.961-0.985) | 0.012 (0.001-0.213) | 1.126 (1.056-1.200) |
| Personality disorders | 0.979 (0.973-0.985) | 0.115 (0.025-0.528) | 1.054 (1.019-1.091) |
| Psychoses | 0.974 (0.968-0.980) | 0.055 (0.010-0.312) | 1.082 (1.039-1.127) |
| **US Open Claims** |  |  |  |
| Depressive disorders | 0.991 (0.990-0.993) | 0.677 (0.501-0.916) | 1.009 (1.003-1.016) |
| Anxiety disorders | 0.992 (0.990-0.993) | 0.720 (0.613-0.846) | 1.010 (1.006-1.013) |
| Alcohol misuse or dependence | 0.994 (0.992-0.995) | 0.824 (0.660-1.028) | 1.004 (1.000-1.009) |
| Substance misuse or dependence | 0.988 (0.987-0.989) | 0.629 (0.511-0.776) | 1.010 (1.005-1.014) |
| Bipolar disorders | 0.988 (0.986-0.989) | 0.687 (0.577-0.818) | 1.010 (1.006-1.014) |
| Personality disorders | 0.996 (0.994-0.998) | 0.698 (0.570-0.856) | 1.008 (1.002-1.013) |
| Psychoses | 0.987 (0.986-0.989) | 0.712 (0.571-0.889) | 1.010 (1.005-1.015) |

**eTable 8. Full estimates from interrupted time-series analyses of the monthly incidence of seven mental health diagnoses**

|  | **Background trend (t)** | **Level change** | **Slope change** |
| --- | --- | --- | --- |
|  | **RR (95% CI)** | **RR (95% CI)** | **RR (95% CI)** |
| **France IQVIA** |  |  |  |
| Depressive disorders | 0.996 (0.993-0.999) | 0.110 (0.074-0.164) | 1.059 (1.049-1.068) |
| Anxiety disorders | 0.995 (0.992-0.998) | 0.196 (0.114-0.336) | 1.046 (1.032-1.059) |
| Alcohol misuse or dependence | 0.996 (0.993-1.000) | 0.120 (0.084-0.173) | 1.055 (1.046-1.064) |
| Substance misuse or dependence | 1.002 (1.000-1.003) | 0.139 (0.099-0.195) | 1.046 (1.039-1.054) |
| Bipolar disorders | 0.995 (0.991-0.999) | 0.281 (0.138-0.574) | 1.037 (1.021-1.054) |
| Personality disorders | 1.000 (0.995-1.005) | 0.102 (0.042-0.251) | 1.060 (1.039-1.081) |
| Psychoses | 0.994 (0.991-0.998) | 0.254 (0.132-0.489) | 1.041 (1.025-1.056) |
| **Germany IQVIA** |  |  |  |
| Depressive disorders | 0.998 (0.996-1.000) | 0.316 (0.253-0.394) | 1.032 (1.027-1.038) |
| Anxiety disorders | 1.005 (1.003-1.006) | 0.351 (0.292-0.422) | 1.028 (1.023-1.033) |
| Alcohol misuse or dependence | 0.996 (0.990-1.001) | 0.339 (0.120-0.962) | 1.030 (1.007-1.055) |
| Substance misuse or dependence | 0.999 (0.995-1.003) | 0.317 (0.155-0.646) | 1.031 (1.014-1.047) |
| Bipolar disorders | 0.994 (0.989-0.998) | 0.224 (0.107-0.470) | 1.043 (1.025-1.060) |
| Personality disorders | 1.004 (1.002-1.006) | 0.393 (0.294-0.525) | 1.024 (1.017-1.031) |
| Psychoses | 1.002 (1.000-1.004) | 0.336 (0.252-0.447) | 1.030 (1.023-1.037) |
| **Italy IQVIA** |  |  |  |
| Depressive disorders | 0.993 (0.990-0.996) | 0.221 (0.160-0.307) | 1.038 (1.031-1.045) |
| Anxiety disorders | 0.994 (0.990-0.998) | 0.208 (0.146-0.298) | 1.040 (1.031-1.049) |
| Alcohol misuse or dependence | 0.990 (0.985-0.995) | 0.199 (0.059-0.666) | 1.040 (1.012-1.069) |
| Substance misuse or dependence | 0.993 (0.988-0.998) | 0.202 (0.104-0.394) | 1.035 (1.020-1.049) |
| Bipolar disorders | 0.997 (0.992-1.003) | 0.381 (0.074-1.961) | 1.021 (0.985-1.058) |
| Personality disorders | 0.997 (0.994-1.001) | 0.058 (0.021-0.158) | 1.066 (1.044-1.089) |
| Psychoses | 0.993 (0.989-0.997) | 0.230 (0.098-0.540) | 1.039 (1.019-1.059) |
| **South Korea AUSOM** |  |  |  |
| Depressive disorders | 1.022 (1.004-1.039) | 1.825 (0.490-6.798) | 0.977 (0.943-1.012) |
| Anxiety disorders | 1.000 (0.996-1.003) | 0.676 (0.235-1.946) | 1.008 (0.985-1.031) |
| Alcohol misuse or dependence | 0.994 (0.976-1.013) | 0.025 (0.001-0.966) | 1.097 (1.012-1.189) |
| Substance misuse or dependence | 0.993 (0.984-1.002) | 0.149 (0.021-1.036) | 1.050 (1.005-1.097) |
| Bipolar disorders | 1.009 (0.998-1.020) | 1.544 (0.163-14.608) | 0.989 (0.940-1.041) |
| Personality disorders | 0.982 (0.963-1.001) | 0.004 (0.000-0.296) | 1.145 (1.042-1.257) |
| Psychoses | 1.002 (0.998-1.005) | 0.800 (0.253-2.535) | 1.005 (0.978-1.032) |
| **South Korea KUN** |  |  |  |
| Depressive disorders | 1.002 (0.998-1.006) | 0.510 (0.230-1.130) | 1.023 (1.004-1.041) |
| Anxiety disorders | 0.996 (0.991-1.001) | 0.377 (0.166-0.860) | 1.029 (1.010-1.047) |
| Alcohol misuse or dependence | 1.005 (1.000-1.009) | 0.377 (0.161-0.884) | 1.025 (1.005-1.046) |
| Substance misuse or dependence | 0.990 (0.987-0.993) | 0.804 (0.252-2.562) | 1.011 (0.985-1.038) |
| Bipolar disorders | 1.005 (0.994-1.015) | 3.173 (0.228-44.251) | 0.976 (0.918-1.037) |
| Personality disorders | 0.996 (0.983-1.009) | 12.717 (0.358-451.429) | 0.955 (0.878-1.038) |
| Psychoses | 1.015 (1.008-1.022) | 0.302 (0.086-1.061) | 1.029 (1.001-1.059) |
| **UK IMRD** |  |  |  |
| Depressive disorders | 0.994 (0.991-0.996) | 0.074 (0.026-0.211) | 1.049 (1.024-1.075) |
| Anxiety disorders | 0.998 (0.997-1.000) | 0.063 (0.032-0.124) | 1.052 (1.036-1.068) |
| Alcohol misuse or dependence | 0.992 (0.989-0.996) | 0.139 (0.060-0.320) | 1.041 (1.022-1.060) |
| Substance misuse or dependence | 0.996 (0.994-0.998) | 0.151 (0.075-0.306) | 1.039 (1.023-1.056) |
| Bipolar disorders | 0.994 (0.990-0.999) | 0.995 (0.234-4.223) | 0.995 (0.963-1.029) |
| Personality disorders | 1.004 (1.000-1.008) | 0.546 (0.160-1.867) | 1.009 (0.980-1.038) |
| Psychoses | 0.994 (0.991-0.998) | 0.958 (0.326-2.821) | 1.000 (0.975-1.025) |
| **US MDCD** |  |  |  |
| Depressive disorders | 0.989 (0.985-0.994) | 0.313 (0.118-0.834) | 1.030 (1.007-1.053) |
| Anxiety disorders | 0.992 (0.986-0.997) | 0.360 (0.123-1.053) | 1.026 (1.002-1.051) |
| Alcohol misuse or dependence | 0.994 (0.988-0.999) | 0.779 (0.260-2.336) | 1.010 (0.985-1.035) |
| Substance misuse or dependence | 0.983 (0.977-0.989) | 0.425 (0.107-1.697) | 1.024 (0.993-1.057) |
| Bipolar disorders | 0.988 (0.983-0.994) | 0.542 (0.161-1.832) | 1.016 (0.988-1.044) |
| Personality disorders | 0.993 (0.989-0.996) | 0.546 (0.253-1.182) | 1.013 (0.995-1.031) |
| Psychoses | 0.988 (0.982-0.994) | 0.563 (0.153-2.072) | 1.014 (0.985-1.045) |
| **US MDCR** |  |  |  |
| Depressive disorders | 0.991 (0.983-0.999) | 0.007 (0.002-0.025) | 1.118 (1.086-1.152) |
| Anxiety disorders | 0.990 (0.981-1.000) | 0.007 (0.001-0.047) | 1.120 (1.075-1.167) |
| Alcohol misuse or dependence | 0.996 (0.989-1.003) | 0.007 (0.003-0.019) | 1.118 (1.092-1.146) |
| Substance misuse or dependence | 0.988 (0.980-0.996) | 0.002 (0.000-0.007) | 1.158 (1.120-1.198) |
| Bipolar disorders | 0.983 (0.970-0.996) | 0.003 (0.000-0.061) | 1.148 (1.077-1.222) |
| Personality disorders | 0.989 (0.984-0.994) | 0.029 (0.013-0.066) | 1.078 (1.058-1.100) |
| Psychoses | 0.985 (0.977-0.992) | 0.016 (0.004-0.068) | 1.103 (1.066-1.141) |
| **US Open Claims** |  |  |  |
| Depressive disorders | 0.993 (0.991-0.994) | 0.332 (0.245-0.450) | 1.026 (1.020-1.032) |
| Anxiety disorders | 0.993 (0.991-0.995) | 0.394 (0.346-0.449) | 1.025 (1.022-1.028) |
| Alcohol misuse or dependence | 0.995 (0.993-0.996) | 0.422 (0.328-0.543) | 1.021 (1.015-1.027) |
| Substance misuse or dependence | 0.989 (0.988-0.991) | 0.309 (0.239-0.400) | 1.027 (1.022-1.032) |
| Bipolar disorders | 0.989 (0.987-0.991) | 0.381 (0.335-0.433) | 1.025 (1.022-1.028) |
| Personality disorders | 0.997 (0.995-0.999) | 0.378 (0.328-0.435) | 1.023 (1.019-1.026) |
| Psychoses | 0.989 (0.987-0.990) | 0.388 (0.338-0.445) | 1.025 (1.022-1.028) |

**eTable 9. Full estimates from sensitivity analysis one: interrupted time-series analyses of the monthly incidence of seven mental health diagnoses using February 2020 as the transition period**

|  | **Background trend (t)** | **Level change** | **Slope change** |
| --- | --- | --- | --- |
|  | **RR (95% CI)** | **RR (95% CI)** | **RR (95% CI)** |
| **France IQVIA** |  |  |  |
| Depressive disorders | 0.996 (0.993-0.999) | 0.116 (0.083-0.162) | 1.058 (1.049-1.066) |
| Anxiety disorders | 0.995 (0.992-0.998) | 0.244 (0.143-0.418) | 1.041 (1.027-1.055) |
| Alcohol misuse or dependence | 0.996 (0.992-1.000) | 0.122 (0.091-0.164) | 1.055 (1.047-1.062) |
| Substance misuse or dependence | 1.002 (1.000-1.004) | 0.163 (0.128-0.206) | 1.043 (1.037-1.048) |
| Bipolar disorders | 0.994 (0.991-0.997) | 0.304 (0.192-0.482) | 1.036 (1.025-1.048) |
| Personality disorders | 0.999 (0.994-1.004) | 0.113 (0.050-0.254) | 1.058 (1.039-1.077) |
| Psychoses | 0.994 (0.990-0.997) | 0.310 (0.171-0.560) | 1.037 (1.023-1.051) |
| **Germany IQVIA** |  |  |  |
| Depressive disorders | 0.998 (0.996-1.000) | 0.330 (0.272-0.400) | 1.031 (1.026-1.036) |
| Anxiety disorders | 1.005 (1.003-1.007) | 0.440 (0.326-0.593) | 1.023 (1.016-1.030) |
| Alcohol misuse or dependence | 0.995 (0.989-1.001) | 0.374 (0.145-0.962) | 1.029 (1.007-1.051) |
| Substance misuse or dependence | 0.999 (0.994-1.004) | 0.337 (0.244-0.464) | 1.029 (1.020-1.038) |
| Bipolar disorders | 0.993 (0.989-0.998) | 0.248 (0.126-0.487) | 1.041 (1.025-1.057) |
| Personality disorders | 1.004 (1.001-1.008) | 0.402 (0.242-0.669) | 1.023 (1.011-1.035) |
| Psychoses | 1.002 (0.999-1.005) | 0.355 (0.232-0.545) | 1.029 (1.018-1.039) |
| **Italy IQVIA** |  |  |  |
| Depressive disorders | 0.993 (0.989-0.996) | 0.176 (0.122-0.255) | 1.043 (1.035-1.051) |
| Anxiety disorders | 0.993 (0.990-0.997) | 0.270 (0.184-0.394) | 1.035 (1.024-1.045) |
| Alcohol misuse or dependence | 0.990 (0.984-0.995) | 0.156 (0.051-0.473) | 1.046 (1.020-1.073) |
| Substance misuse or dependence | 0.992 (0.988-0.995) | 0.162 (0.101-0.261) | 1.041 (1.030-1.051) |
| Bipolar disorders | 0.998 (0.993-1.003) | 0.404 (0.126-1.303) | 1.019 (0.994-1.045) |
| Personality disorders | 0.997 (0.994-1.001) | 0.040 (0.014-0.113) | 1.075 (1.052-1.098) |
| Psychoses | 0.993 (0.989-0.997) | 0.251 (0.116-0.540) | 1.037 (1.019-1.056) |
| **South Korea AUSOM** |  |  |  |
| Depressive disorders | 1.022 (1.005-1.040) | 2.688 (0.584-12.385) | 0.968 (0.928-1.010) |
| Anxiety disorders | 1.001 (0.998-1.003) | 0.655 (0.290-1.478) | 1.008 (0.990-1.026) |
| Alcohol misuse or dependence | 0.993 (0.974-1.012) | 0.045 (0.002-1.171) | 1.085 (1.008-1.168) |
| Substance misuse or dependence | 0.993 (0.983-1.002) | 0.230 (0.040-1.311) | 1.040 (1.000-1.083) |
| Bipolar disorders | 1.010 (0.998-1.021) | 1.232 (0.163-9.294) | 0.993 (0.948-1.041) |
| Personality disorders | 0.982 (0.962-1.002) | 0.017 (0.000-0.766) | 1.111 (1.020-1.209) |
| Psychoses | 1.002 (0.997-1.008) | 2.134 (0.584-7.801) | 0.983 (0.954-1.014) |
| **South Korea KUN** |  |  |  |
| Depressive disorders | 1.003 (0.999-1.007) | 0.457 (0.223-0.935) | 1.025 (1.008-1.042) |
| Anxiety disorders | 0.995 (0.990-1.000) | 0.269 (0.127-0.572) | 1.037 (1.020-1.054) |
| Alcohol misuse or dependence | 1.006 (1.000-1.012) | 0.400 (0.202-0.793) | 1.023 (1.006-1.040) |
| Substance misuse or dependence | 0.989 (0.986-0.992) | 0.392 (0.098-1.569) | 1.028 (0.995-1.061) |
| Bipolar disorders | 1.005 (0.993-1.017) | 1.804 (0.158-20.607) | 0.987 (0.933-1.044) |
| Personality disorders | 0.995 (0.974-1.017) | 4.924 (0.124-196.290) | 0.975 (0.894-1.065) |
| Psychoses | 1.015 (1.008-1.022) | 0.348 (0.113-1.077) | 1.026 (1.000-1.053) |
| **UK IMRD** |  |  |  |
| Depressive disorders | 0.994 (0.990-0.998) | 0.541 (0.151-1.940) | 1.004 (0.974-1.035) |
| Anxiety disorders | 0.998 (0.995-1.001) | 0.176 (0.067-0.461) | 1.028 (1.005-1.052) |
| Alcohol misuse or dependence | 0.991 (0.987-0.995) | 0.181 (0.058-0.569) | 1.036 (1.009-1.064) |
| Substance misuse or dependence | 0.996 (0.992-0.999) | 0.208 (0.081-0.535) | 1.032 (1.010-1.055) |
| Bipolar disorders | 0.993 (0.989-0.998) | 1.701 (0.503-5.752) | 0.985 (0.957-1.014) |
| Personality disorders | 1.003 (0.999-1.008) | 0.757 (0.257-2.230) | 1.002 (0.976-1.027) |
| Psychoses | 0.994 (0.989-0.998) | 1.446 (0.998-2.095) | 0.991 (0.983-0.999) |
| **US MDCD** |  |  |  |
| Depressive disorders | 0.988 (0.983-0.992) | 0.386 (0.165-0.899) | 1.026 (1.007-1.046) |
| Anxiety disorders | 0.990 (0.985-0.995) | 0.443 (0.173-1.135) | 1.023 (1.001-1.045) |
| Alcohol misuse or dependence | 0.992 (0.986-0.997) | 0.872 (0.339-2.244) | 1.009 (0.987-1.031) |
| Substance misuse or dependence | 0.981 (0.975-0.987) | 0.550 (0.166-1.815) | 1.020 (0.993-1.049) |
| Bipolar disorders | 0.987 (0.981-0.993) | 0.683 (0.235-1.984) | 1.012 (0.987-1.037) |
| Personality disorders | 0.993 (0.989-0.997) | 0.730 (0.548-0.973) | 1.007 (0.999-1.015) |
| Psychoses | 0.987 (0.981-0.993) | 0.705 (0.224-2.219) | 1.011 (0.984-1.038) |
| **US MDCR** |  |  |  |
| Depressive disorders | 0.992 (0.983-1.000) | 0.007 (0.001-0.033) | 1.120 (1.080-1.160) |
| Anxiety disorders | 0.992 (0.982-1.001) | 0.009 (0.002-0.047) | 1.115 (1.073-1.158) |
| Alcohol misuse or dependence | 0.997 (0.989-1.005) | 0.006 (0.001-0.026) | 1.120 (1.085-1.156) |
| Substance misuse or dependence | 0.988 (0.979-0.998) | 0.002 (0.000-0.010) | 1.158 (1.112-1.205) |
| Bipolar disorders | 0.987 (0.974-1.000) | 0.004 (0.000-0.043) | 1.143 (1.082-1.209) |
| Personality disorders | 0.991 (0.986-0.996) | 0.027 (0.014-0.052) | 1.078 (1.062-1.095) |
| Psychoses | 0.985 (0.975-0.996) | 0.013 (0.002-0.089) | 1.106 (1.060-1.154) |
| **US Open Claims** |  |  |  |
| Depressive disorders | 0.993 (0.991-0.994) | 0.330 (0.258-0.423) | 1.026 (1.021-1.032) |
| Anxiety disorders | 0.993 (0.991-0.995) | 0.392 (0.352-0.437) | 1.025 (1.023-1.028) |
| Alcohol misuse or dependence | 0.995 (0.993-0.996) | 0.414 (0.344-0.497) | 1.021 (1.018-1.025) |
| Substance misuse or dependence | 0.989 (0.987-0.991) | 0.326 (0.275-0.386) | 1.026 (1.022-1.029) |
| Bipolar disorders | 0.989 (0.987-0.991) | 0.384 (0.347-0.424) | 1.025 (1.022-1.027) |
| Personality disorders | 0.997 (0.995-0.999) | 0.383 (0.345-0.426) | 1.022 (1.019-1.025) |
| Psychoses | 0.988 (0.987-0.990) | 0.387 (0.346-0.433) | 1.025 (1.023-1.028) |

**eTable 10. Full estimates from sensitivity analysis two: interrupted time-series analyses of the monthly incidence of seven mental health diagnoses using March to April 2022 as the transition period**

|  | **Background trend (t)** | **Level change** | **Slope change** |
| --- | --- | --- | --- |
|  | **RR (95% CI)** | **RR (95% CI)** | **RR (95% CI)** |
| **France IQVIA** |  |  |  |
| Depressive disorders | 0.996 (0.993-0.999) | 0.150 (0.085-0.264) | 1.053 (1.039-1.067) |
| Anxiety disorders | 0.995 (0.992-0.998) | 0.191 (0.102-0.284) | 1.047 (1.031-1.064) |
| Alcohol misuse or dependence | 0.997 (0.993-1.001) | 0.178 (0.107-0.297) | 1.047 (1.034-1.060) |
| Substance misuse or dependence | 1.002 (1.000-1.003) | 0.207 (0.147-0.291) | 1.039 (1.030-1.047) |
| Bipolar disorders | 0.995 (0.991-0.999) | 0.270 (0.142-0.516) | 1.039 (1.023-1.060) |
| Personality disorders | 1.000 (0.997-1.002) | 0.154 (0.111-0.214) | 1.052 (1.045-1.060) |
| Psychoses | 0.994 (0.991-0.998) | 0.219 (0.107-0.448) | 1.045 (1.028-1.062) |
| **Germany IQVIA** |  |  |  |
| Depressive disorders | 0.998 (0.996-1.001) | 0.365 (0.276-0.482) | 1.030 (1.022-1.037) |
| Anxiety disorders | 1.005 (1.003-1.006) | 0.376 (0.291-0.486) | 1.027 (1.021-1.034) |
| Alcohol misuse or dependence | 0.996 (0.988-1.004) | 0.404 (0.206-0.793) | 1.027 (1.010-1.045) |
| Substance misuse or dependence | 0.999 (0.994-1.004) | 0.344 (0.219-0.541) | 1.029 (1.018-1.041) |
| Bipolar disorders | 0.994 (0.989-0.998) | 0.253 (0.114-0.563) | 1.041 (1.022-1.060) |
| Personality disorders | 1.004 (1.003-1.006) | 0.430 (0.324-0.573) | 1.022 (1.015-1.029) |
| Psychoses | 1.002 (1.000-1.004) | 0.349 (0.244-0.499) | 1.030 (1.021-1.039) |
| **Italy IQVIA** |  |  |  |
| Depressive disorders | 0.993 (0.990-0.997) | 0.331 (0.226-0.486) | 1.030 (1.020-1.039) |
| Anxiety disorders | 0.994 (0.990-0.998) | 0.295 (0.165-0.528) | 1.033 (1.018-1.048) |
| Alcohol misuse or dependence | 0.990 (0.985-0.995) | 0.391 (0.113-1.352) | 1.026 (0.997-1.055) |
| Substance misuse or dependence | 0.993 (0.989-0.998) | 0.429 (0.244-0.755) | 1.019 (1.004-1.034) |
| Bipolar disorders | 0.997 (0.992-1.002) | 0.977 (0.262-3.635) | 1.001 (0.971-1.032) |
| Personality disorders | 0.998 (0.995-1.001) | 0.142 (0.065-0.309) | 1.047 (1.028-1.066) |
| Psychoses | 0.993 (0.989-0.997) | 0.273 (0.107-0.694) | 1.036 (1.014-1.058) |
| **South Korea AUSOM** |  |  |  |
| Depressive disorders | 1.022 (1.005-1.039) | 1.349 (0.338-5.379) | 0.983 (0.949-1.019) |
| Anxiety disorders | 1.000 (0.996-1.003) | 0.299 (0.153-0.585) | 1.026 (1.011-1.042) |
| Alcohol misuse or dependence | 0.994 (0.976-1.012) | 0.080 (0.002-3.290) | 1.073 (0.986-1.167) |
| Substance misuse or dependence | 0.993 (0.986-1.001) | 0.288 (0.079-1.042) | 1.035 (1.003-1.069) |
| Bipolar disorders | 1.009 (0.998-1.020) | 2.130 (0.179-25.280) | 0.982 (0.928-1.040) |
| Personality disorders | 0.982 (0.963-1.001) | 0.003 (0.000-0.306) | 1.157 (1.043-1.284) |
| Psychoses | 1.002 (0.993-1.011) | 1.063 (0.123-9.186) | 0.998 (0.950-1.049) |
| **South Korea KUN** |  |  |  |
| Depressive disorders | 1.002 (0.998-1.006) | 0.708 (0.300-1.667) | 1.016 (0.996-1.036) |
| Anxiety disorders | 0.995 (0.990-1.000) | 0.342 (0.104-1.124) | 1.032 (1.004-1.060) |
| Alcohol misuse or dependence | 1.005 (1.000-1.009) | 0.462 (0.183-1.169) | 1.021 (1.000-1.043) |
| Substance misuse or dependence | 0.990 (0.984-0.996) | 1.078 (0.171-6.805) | 1.005 (0.962-1.049) |
| Bipolar disorders | 1.005 (0.995-1.015) | 9.855 (0.887-109.525) | 0.951 (0.898-1.007) |
| Personality disorders | 0.994 (0.973-1.015) | 53.222 (0.587-4845.356) | 0.925 (0.831-1.030) |
| Psychoses | 1.015 (1.008-1.022) | 0.241 (0.060-0.965) | 1.035 (1.003-1.069) |
| **UK IMRD** |  |  |  |
| Depressive disorders | 0.993 (0.992-0.995) | 0.163 (0.100-0.268) | 1.032 (1.020-1.044) |
| Anxiety disorders | 0.998 (0.997-1.000) | 0.107 (0.067-0.170) | 1.041 (1.030-1.052) |
| Alcohol misuse or dependence | 0.992 (0.989-0.996) | 0.330 (0.213-0.513) | 1.022 (1.011-1.032) |
| Substance misuse or dependence | 0.996 (0.994-0.998) | 0.313 (0.211-0.464) | 1.023 (1.013-1.033) |
| Bipolar disorders | 0.994 (0.990-0.999) | 0.886 (0.188-4.166) | 0.998 (0.963-1.035) |
| Personality disorders | 1.004 (1.000-1.008) | 0.860 (0.221-3.339) | 0.999 (0.967-1.031) |
| Psychoses | 0.994 (0.991-0.998) | 1.095 (0.321-3.737) | 0.997 (0.968-1.026) |
| **US MDCD** |  |  |  |
| Depressive disorders | 0.989 (0.985-0.994) | 0.389 (0.134-1.133) | 1.025 (1.001-1.051) |
| Anxiety disorders | 0.992 (0.986-0.997) | 0.423 (0.130-1.377) | 1.023 (0.996-1.051) |
| Alcohol misuse or dependence | 0.994 (0.988-0.999) | 0.974 (0.292-3.249) | 1.005 (0.978-1.033) |
| Substance misuse or dependence | 0.984 (0.974-0.994) | 0.760 (0.314-1.841) | 1.011 (0.985-1.038) |
| Bipolar disorders | 0.988 (0.983-0.994) | 0.578 (0.150-2.229) | 1.015 (0.984-1.046) |
| Personality disorders | 0.993 (0.989-0.996) | 0.607 (0.259-1.423) | 1.011 (0.991-1.031) |
| Psychoses | 0.988 (0.982-0.994) | 0.611 (0.144-2.587) | 1.013 (0.980-1.047) |
| **US MDCR** |  |  |  |
| Depressive disorders | 0.991 (0.983-0.999) | 0.010 (0.003-0.042) | 1.112 (1.075-1.151) |
| Anxiety disorders | 0.991 (0.983-1.000) | 0.012 (0.003-0.048) | 1.110 (1.073-1.148) |
| Alcohol misuse or dependence | 0.996 (0.989-1.003) | 0.009 (0.003-0.029) | 1.116 (1.084-1.148) |
| Substance misuse or dependence | 0.988 (0.980-0.997) | 0.002 (0.000-0.012) | 1.153 (1.109-1.198) |
| Bipolar disorders | 0.983 (0.970-0.996) | 0.004 (0.000-0.093) | 1.147 (1.069-1.231) |
| Personality disorders | 0.989 (0.983-0.996) | 0.034 (0.006-0.181) | 1.076 (1.037-1.117) |
| Psychoses | 0.985 (0.977-0.993) | 0.019 (0.003-0.109) | 1.101 (1.056-1.148) |
| **US Open Claims** |  |  |  |
| Depressive disorders | 0.993 (0.991-0.994) | 0.414 (0.358-0.478) | 1.022 (1.019-1.025) |
| Anxiety disorders | 0.993 (0.991-0.995) | 0.438 (0.394-0.488) | 1.023 (1.020-1.026) |
| Alcohol misuse or dependence | 0.995 (0.993-0.996) | 0.507 (0.464-0.554) | 1.017 (1.015-1.019) |
| Substance misuse or dependence | 0.989 (0.988-0.990) | 0.386 (0.348-0.428) | 1.023 (1.020-1.025) |
| Bipolar disorders | 0.989 (0.987-0.991) | 0.421 (0.381-0.465) | 1.023 (1.020-1.026) |
| Personality disorders | 0.997 (0.995-0.999) | 0.426 (0.393-0.461) | 1.021 (1.018-1.023) |
| Psychoses | 0.989 (0.987-0.990) | 0.437 (0.405-0.472) | 1.023 (1.021-1.025) |

**eFigure 1. COVID-19 Stringency Index between January 2020 and January 2022 in France, Germany, Italy, South Korea, the UK, and the US**


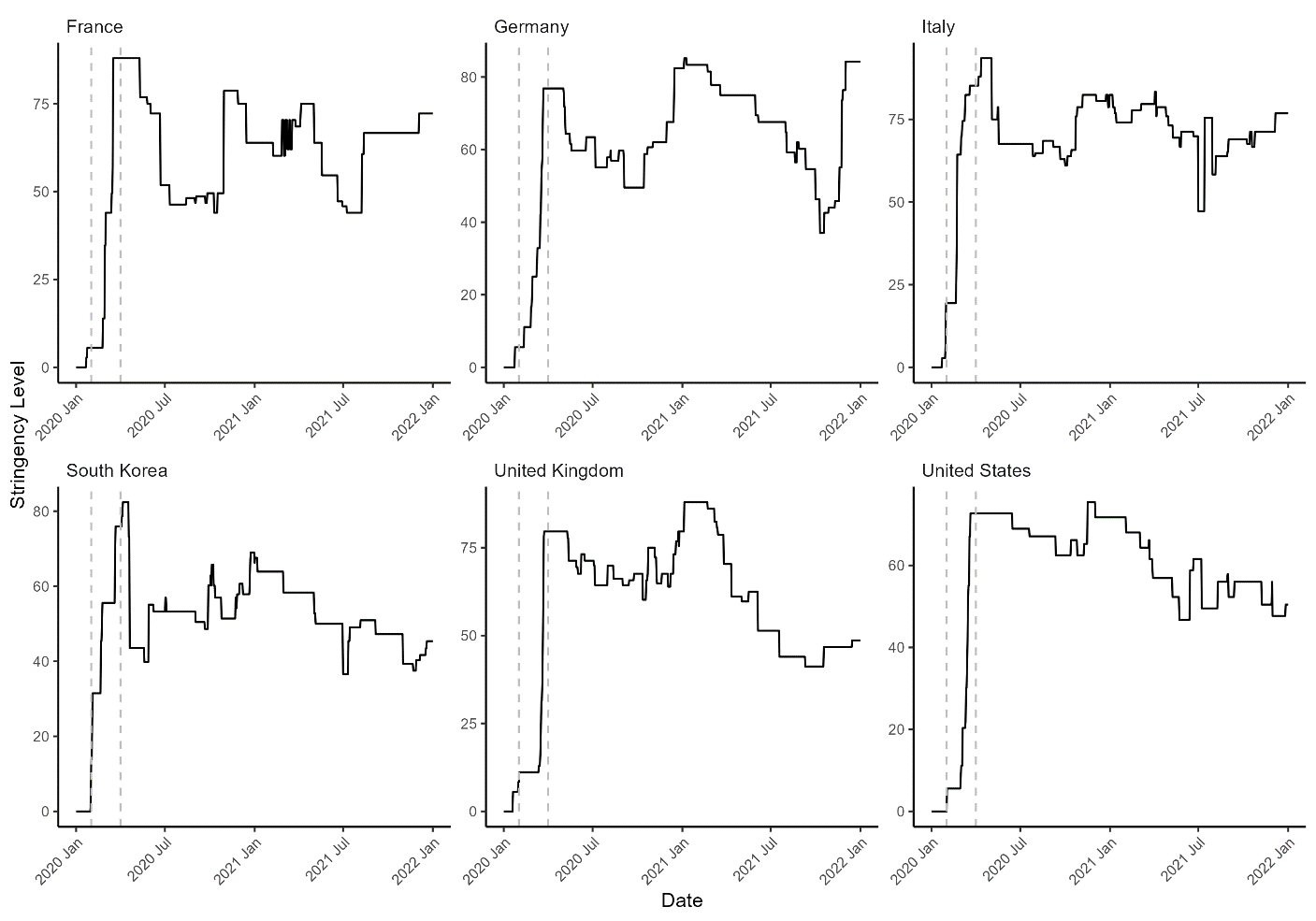


Vertical dashed lines represent February and April 2020

**eFigure 2. Interrupted series analysis of changes in monthly number of incident cases of seven mental health diagnoses**


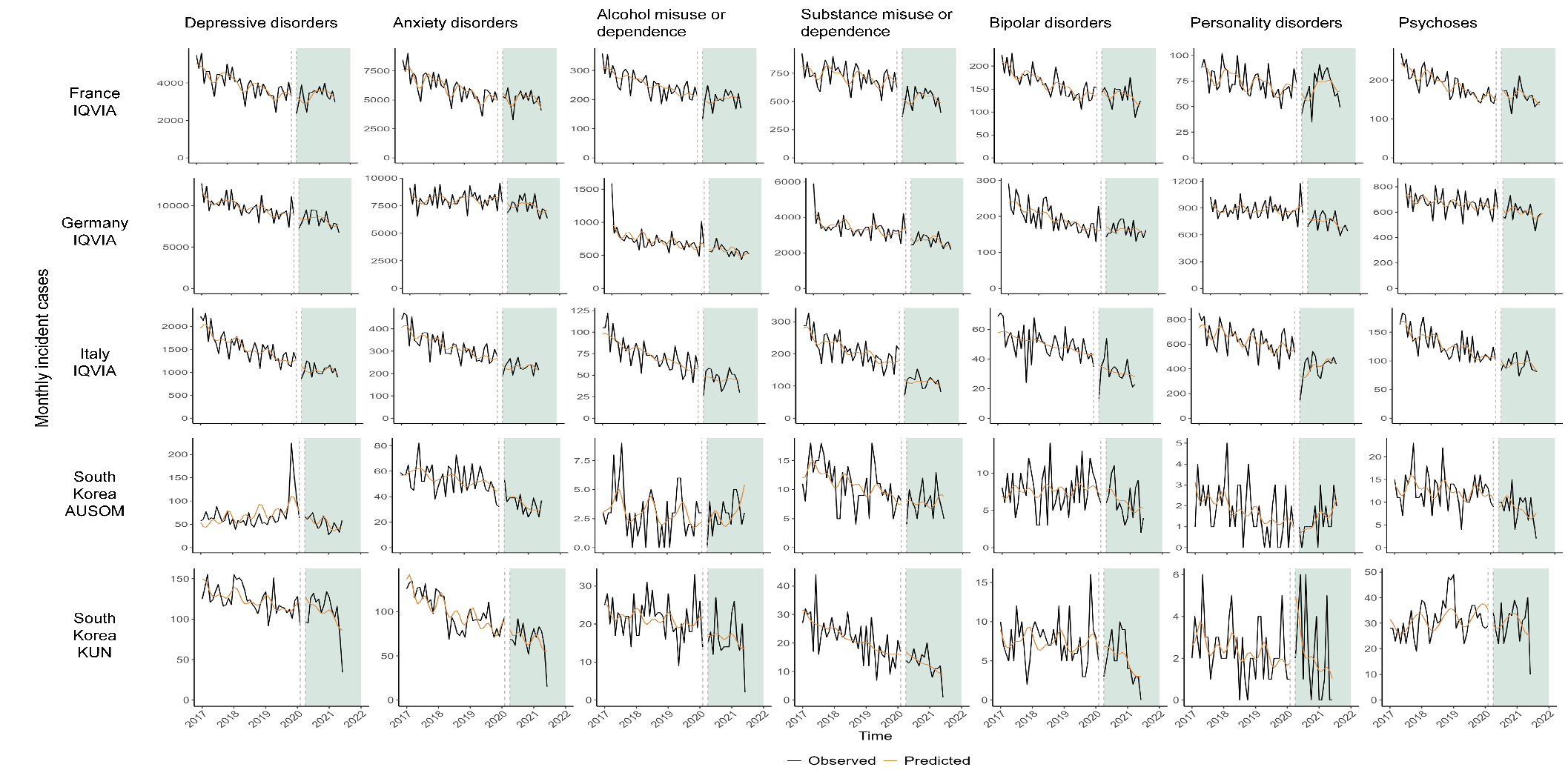


Vertical dashed lines represent February and April 2020

Green area represents the period after the introduction of national lockdown restrictions and containment strategies

Black lines represent observed trends

Orange lines represent predicted trends based on the ITS model

**eFigure 2. *continued.* Interrupted series analysis of changes in monthly number of incident cases of seven mental health diagnoses**


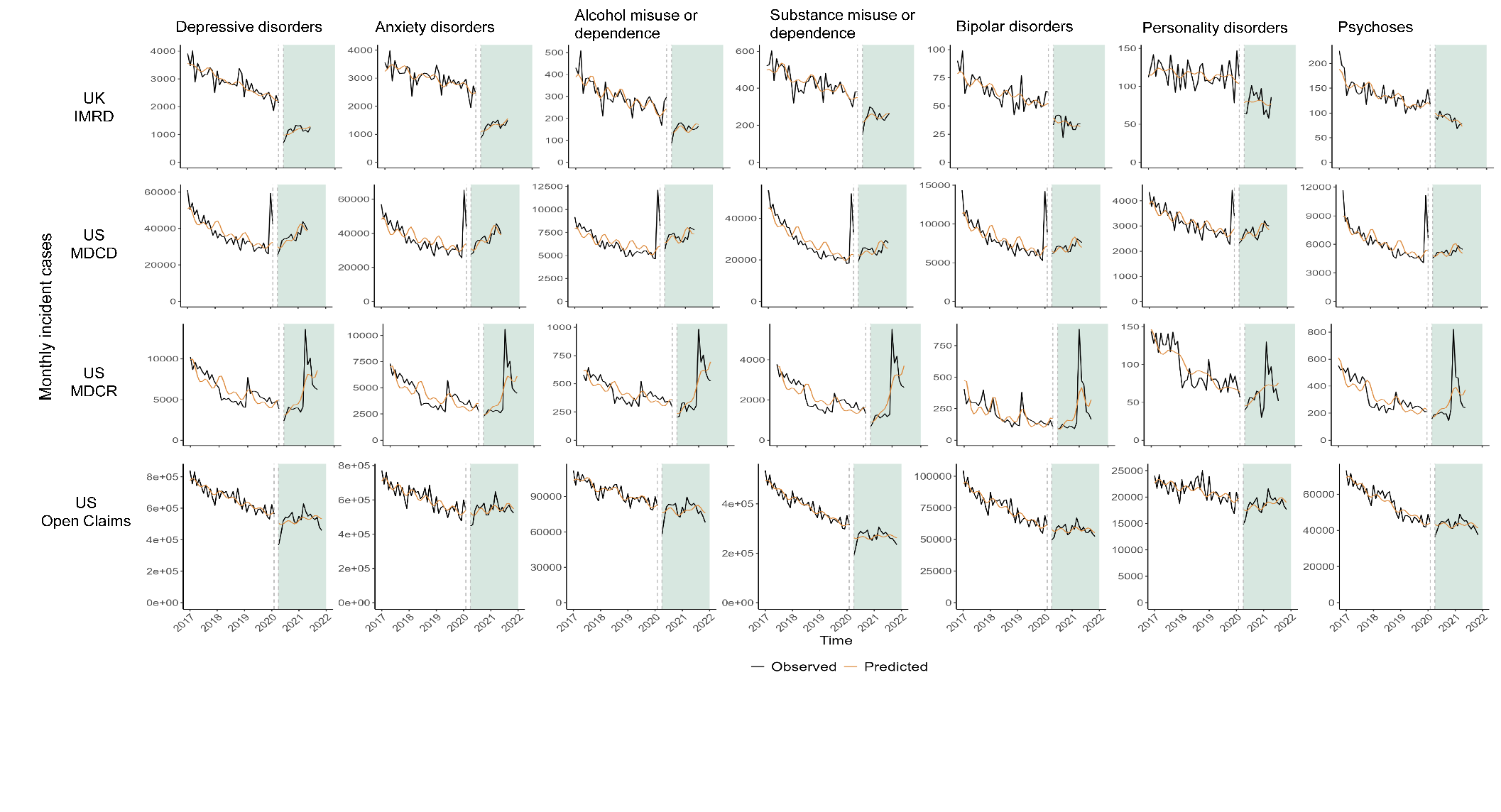


Vertical dashed lines represent February and April 2020

Green area represents the period after the introduction of national lockdown restrictions and containment strategies

Black lines represent observed trends

Orange lines represent predicted trends based on the ITS model

**eFigure 3. Interrupted series analysis of changes in monthly incidence of seven mental health diagnoses**


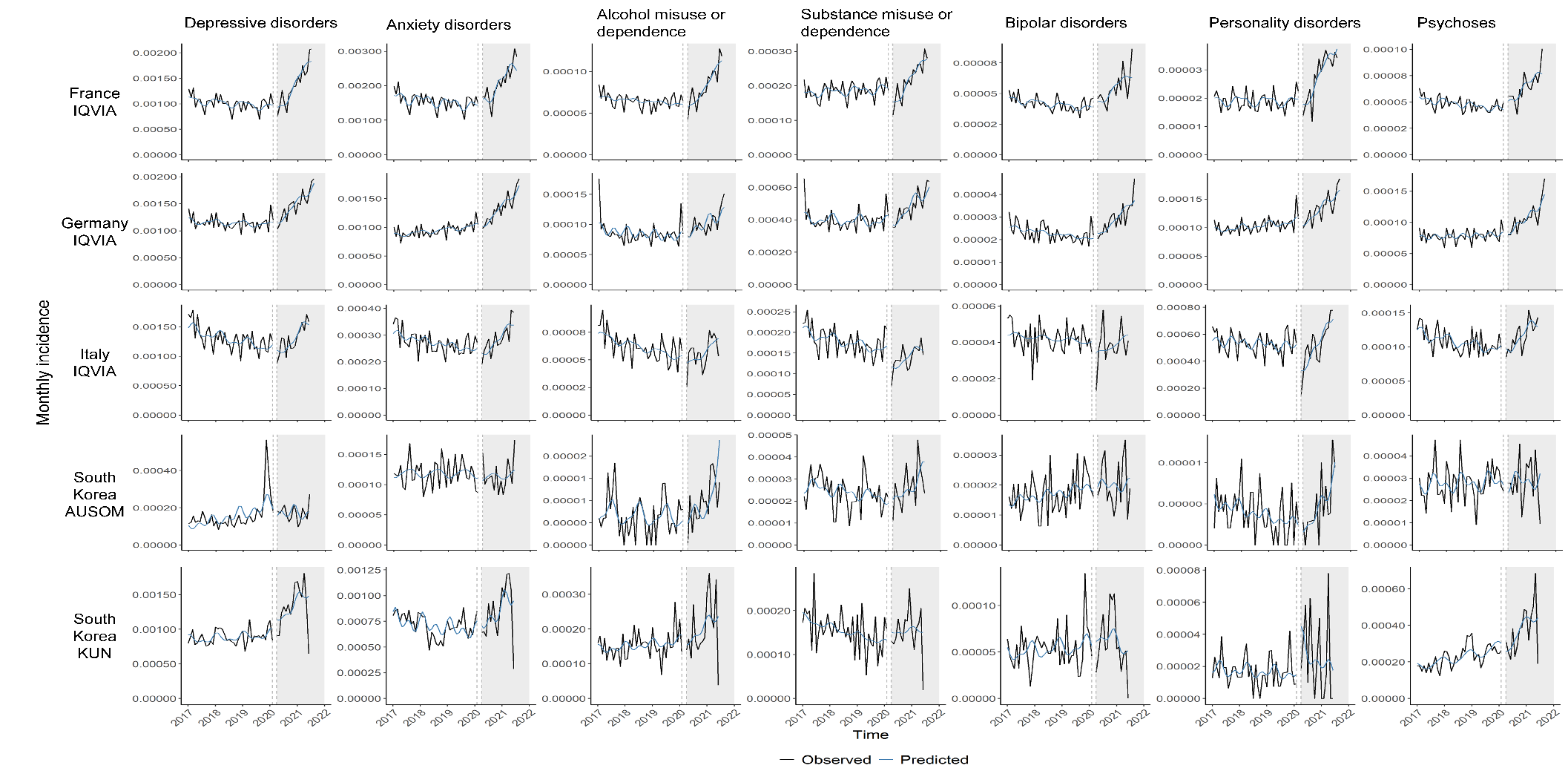


Vertical dashed lines represent February and April 2020

Grey area represents the period after the introduction of national lockdown restrictions and containment strategies

Black lines represent observed trends

Blue lines represent predicted trends based on the ITS model

**eFigure 3. *continued.* Interrupted series analysis of changes in monthly incidence of seven mental health diagnoses**


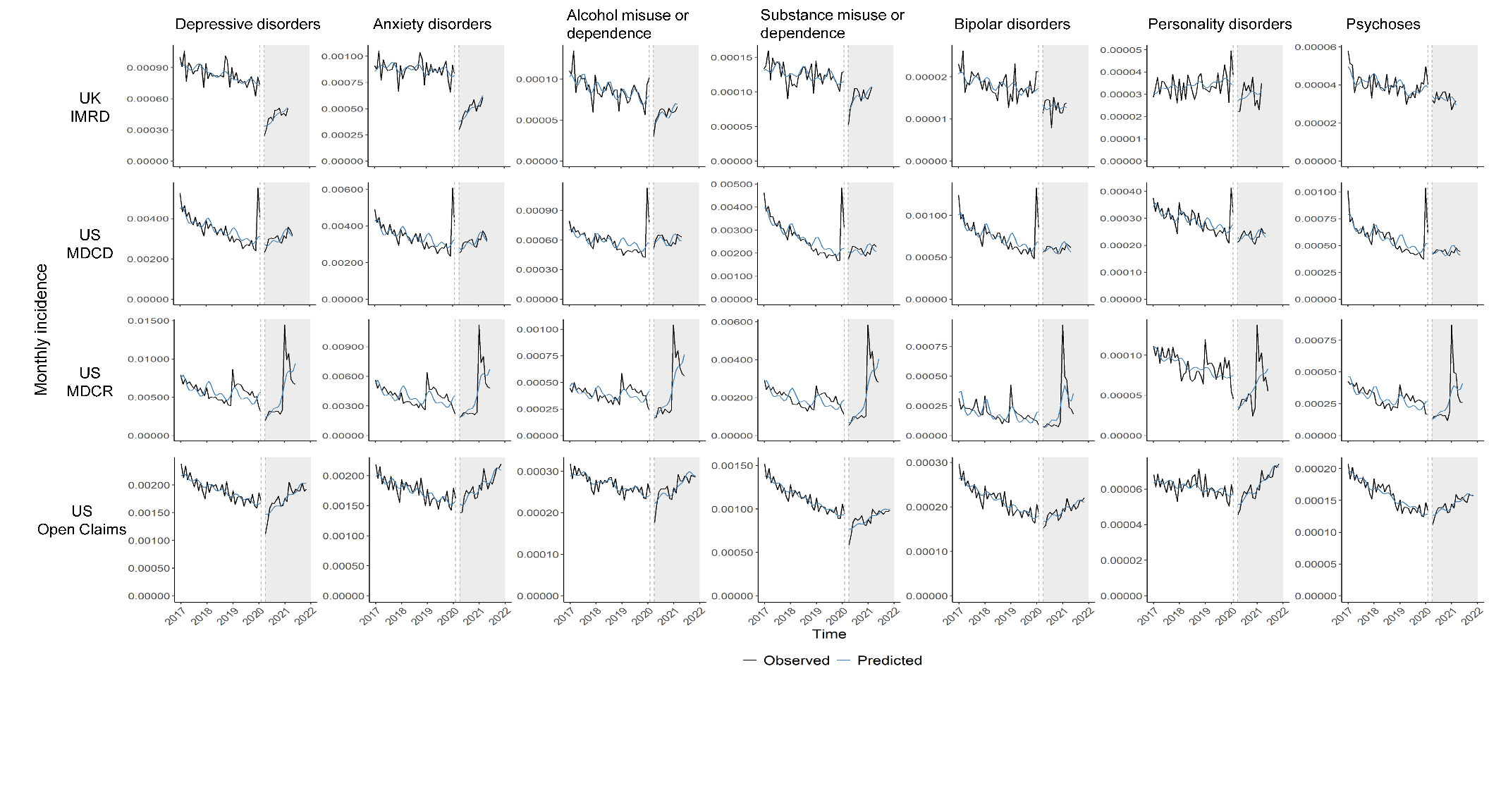


Vertical dashed lines represent February and April 2020

Grey area represents the period after the introduction of national lockdown restrictions and containment strategies

Black lines represent observed trends

Blue lines represent predicted trends based on the ITS model
